# Magnetic Particles Coupled to Disposable Screen Printed Transducers for Electrochemical Biosensing

**DOI:** 10.3390/s16101585

**Published:** 2016-09-25

**Authors:** Paloma Yáñez-Sedeño, Susana Campuzano, José M. Pingarrón

**Affiliations:** Departamento de Química Analítica, Facultad de CC. Químicas, Universidad Complutense de Madrid, E-28040 Madrid, Spain; susanacr@quim.ucm.es (S.C.); pingarro@quim.ucm.es (J.M.P.)

**Keywords:** magnetic materials, screen-printed electrodes, electrochemical affinity biosensors

## Abstract

Ultrasensitive biosensing is currently a growing demand that has led to the development of numerous strategies for signal amplification. In this context, the unique properties of magnetic particles; both of nano- and micro-size dimensions; have proved to be promising materials to be coupled with disposable electrodes for the design of cost-effective electrochemical affinity biosensing platforms. This review addresses, through discussion of selected examples, the way that nano- and micro-magnetic particles (MNPs and MMPs; respectively) have contributed significantly to the development of electrochemical affinity biosensors, including immuno-, DNA, aptamer and other affinity modes. Different aspects such as type of magnetic particles, assay formats, detection techniques, sensitivity, applicability and other relevant characteristics are discussed. Research opportunities and future development trends in this field are also considered.

## 1. Introduction

Applications of superparamagnetic iron oxide particles with sizes between a few nanometers and micrometers units are numerous and extend to fields as varied as clinical analysis, food and environmental safety, drug delivery, or molecular imaging. The possibility of easy functionalization with diverse reactive moieties such as carboxyl, amine, aldehyde, hydroxyl, thiol or tosyl), as well as the ability for interact with biomolecules (e.g., antibodies, oligonucleotides, protein A or G, streptavidin), and the development of efficient methods for the synthesis of magnetic nanoparticles with improved properties when associated with different materials, makes them a useful and versatile tool to recognize a variety of targets. Moreover, the magnetic properties of these particles allow enhancing the isolation and preconcentration of the analyte from complex samples, and providing a useful technology by implementing various types of magnetic devices including biosensors or microfluidic platforms among others.

Electrochemical affinity biosensors are usually prepared by immobilization of the bioreceptor onto an electrode surface whose selection is crucial to achieve an efficient and stable incorporation of the recognition element, as well as a high sensitivity. The development of these biosensors typically requires various steps and several calibration points. Moreover, small sample volumes are analyzed. These reasons make screen printed electrodes (SPEs) highly attractive and preferable over other types of electrodes for such applications [1]. SPEs are suitable to be fabricated with different materials, to be drawn in diverse geometries and arrays [2], and characterized by the possibility for mass production and low fabrication cost. In addition, the small size of these electrodes enables drops to be used for the assays and, importantly in the context of this review, the planar shape of SPEs facilitates the incorporation of magnetic bioconjugates by simple attraction using a magnet positioned under the electrode.

Coupling of electrochemical transduction with the use of magnetic nano/microcarriers has greatly contributed to significant improvements in the performance of electrochemical biosensors. The application of superparamagnetic particles based on iron oxide functionalized with diverse reactive groups for the easy, fast and selective capture of a specific target molecule from a complex sample, and further coupling with disposable electrochemical sensors, is nowadays a well-established methodology [3]. These particles enable the efficient immobilization of capture bioreceptors, allow faster assay kinetics, minimize unspecific adsorptions frequently occurring in complex samples, and avoid the need for delicate electrode preparation to enable control of density and orientation of recognition probes at the disposable electrode surface [4]. Other applications take advantage also of the easy handling, modification and manipulation of these magnetic particles for the preparation of magnetic bioconjugates useful as advanced labels for amplification signal purposes.

This review discusses briefly the main characteristics and advantages offered by micro- and nano-magnetic particles in electrochemical biosensing making special emphasis on recent advances in the field of electrochemical affinity platforms involving the coupling of these magnetic materials and disposable electrochemical transducers. The highlighted approaches are classified according to the size of the magnetic particles used, as well as to its use as electrode modifiers or carriers of signaling molecules and type of biosensing platform. Single- or multiplexed capabilities of the magnetic particles-based disposable biaffinity platforms are also differentiated. In addition, the bottlenecks and possible research directions are also pointed out.

## 2. Micromagnetic Particles

Micro-magnetic particles (MMPs) consisted of a dispersion of Fe_2_O_3_ and Fe_3_O_4_ magnetic material coated by a polymeric thin shell that define also a surface area for interaction by coupling or adsorption with a variety of molecules.

Currently there are commercially available MMPs modified with various functional groups (tosyl, amine, carboxyl or epoxy) and ligands such as antibodies, nucleic acids, proteins or small molecules for specific applications. Hence, MMPs can be tailored-modified in an easy way with a whole range of ligands in bioaffinity reactions [5].

These highly reactive materials of relatively low volume constitute a versatile tool in the development of biosensors, as they exhibit a large surface area for binding in an oriented way recognition elements or specific analytes, which can be easily isolated from the solution with a small magnet and promptly redispersed when the magnet is placed out. In addition to significantly accelerate the kinetics of the recognition processes, the reagents that are not specifically linked or unwanted sample components can be removed easily and efficiently by magnetically controlled washing, thus giving them utility for purification and preconcentration purposes and suitability for automation [6]. The use of MMPs greatly facilitates the preparation of biosensors and detection steps [7], leading to strategies that generally exhibit better detection limits (LODs) and lower matrix effects than conventional integrated configurations [8,9]. Additional advantages of the use of MMPs are the small sample volumes of solution required for their modification, the large quantity of recognition element that can be immobilized in a single step, and that MMPs bioconjugates can be stored for several weeks without loss of activity. This last advantage is crucial when a large number of samples have to be analyzed, and could lead to a meaningful reduction of the analysis time.

Probably, the major drawback associated with the use of MMPs-based electrochemical sensors is the lack of uncomplicated, low cost and practical magnetic electrodes for MMPs manipulation. Direct sensing has been appropriately solved by using planar disposable electrodes with small magnets placed below their surface. This makes it possible to concentrate MMPs on the active area of the working electrode in the best conditions for the electrochemical measurement once the affinity reactions took place [1,10].

The most important characteristics of the electrochemical bioaffinity sensors involving the use of MMPs and disposable electrodes, discussed in Section 2.1, Section 2.2, Section 2.3 and Section 2.4, are summarized in Tables S1–S3 (in the Supporting Information).

### 2.1. Magnetic Microparticles-Screen Printed Electrodeselectrochemical Immunosensors

Moreno-Guzmán et al. [11] developed an electrochemical immunosensor based on MMPs and screen-printed carbon electrodes (SPCEs) for the determination of cortisol, an important stress biomarker of great relevance in sport medicine. The specific anti-cortisol antibody was immobilized onto Protein A (Prot A)-MMPs, and cortisol quantification was implemented by a direct competitive immunoassay involving cortisol antigen labeled with alkaline phosphatase (AP) and 1-naphthyl phosphate as AP substrate. Differential pulse voltammetry (DPV) was the electrochemical technique used to detect the generated 1-naphthol. A calibration plot with a linear range between 5.0 × 10^−3^ and 150 ng·mL^−1^ cortisol, and a LOD of 3.5 pg·mL^−1^ were obtained. The usefulness of this immunosensing approach was demonstrated by application to certified human sera containing cortisol at two different concentration levels.

The same group developed also a similar immunosensing approach for testosterone with horseradish peroxidase (HRP)-labeled antigen and amperometric detection at SCPEs using the system H_2_O_2_/HQ [12]. This immunosensor exhibited a linear calibration plot between 5.0 × 10^−3^ and 50 ng·mL^−1^ testosterone and a LOD of 1.7 pg·mL^−1^, and was successfully applied to the determination of the hormone in spiked serum.

With the objective of specifically detect *Streptococcus pneumoniae*, disposable amperometric magnetoimmunosensors using ProtA-MMPs, and a sandwich format with the same antibody (produced in the laboratory) used unconjugated as capture (Ab) and HRP-labeled as detector (HRP-Ab), were reported. Amperometric detection of H_2_O_2_ at tetrathiafulvalene (TTF)-modified Au-SPEs was employed [13]. LODs of 1.510^4^ and 6.3 × 10^5^ colony forming unit (cfu) mL^−1^ for model capsulated (Dawn) and non-encapsulated (R6) *Streptococcus pneumoniae* selected strains, were achieved, respectively, with no need for pre-concentration or pre-enrichment steps. Results demonstrated a good selectivity of the immunosensor against other closely related streptococci, and the analytical utility of the developed methodology for application to inoculated urine.

A sandwich immunoassay was developed for myoglobin (a protein marker for muscle damage) using immunoconjugates of silver nanoparticles as labels and immunoconjugates of MMPs as microcarriers for selective capture of the target protein [14]. The sandwich immunoconjugates where myoglobin was linked to both tosyl activated-MMPs and silver nanoparticles were magnetically captured on the surface of SPCEs. To perform the electrochemical transduction, ammonium thiocyanate was added to break the captured conjugates and stabilize silver. Finally, the silver nanoparticles were dissolved to form silver ions and, after nucleation and accumulation steps of silver on the electrode surface, the stripping voltammetric signal corresponding to silver removal from the electrode surface was recorded and related to the myoglobin concentration. This approach provided a dynamic range ranging between 0.2–20 ng·mL^−1^ (10 pM–1 nM), far below the normal human serum myoglobin levels (30–90 ng·mL^−1^) and the elevated levels found within an hour of onset of myocardial infarction (200–900 ng·mL^−1^).

Moreno-Guzmán et al. [15] designed a sandwich immunoassay for prolactin (PRL), a relevant hormone in lactation and reproduction, using streptavidin (Strep)-MMPs, SPCEs and a detector antibody labeled with AP. Prolactin quantification was accomplished by DPV of generated 1-naphtol by hydrolysis of 1-naphtyl phosphate in the presence of AP. A linear range between 10 and 2000 ng·mL^−1^ prolactin (slope value of 7.0 nA·mL ng^−1^) and a LOD of 3.74 ng·mL^−1^ were found. The applicability of this approach was demonstrated by analyzing spiked certified human serum.

**Figure 1:**
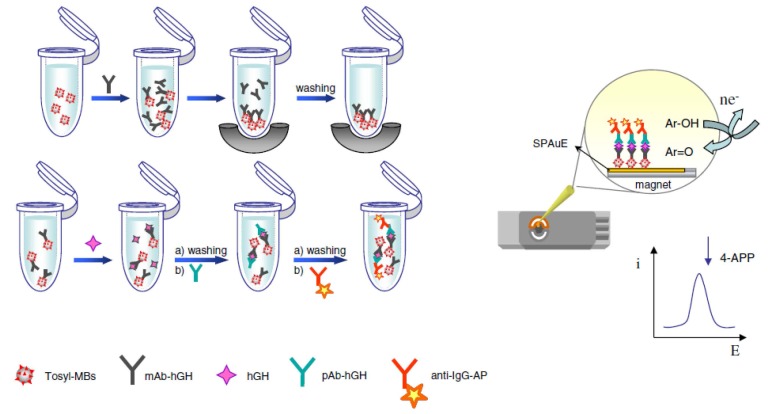
Scheme of the preparation and functioning of the disposable magneto-immunosensor developed for the determination of human growth hormone (hGH). Reprinted from Ref. [16] with permission.

One year after, this same group prepared an electrochemical magnetoimmunosensor for the detection of human growth hormone (hGH) [16] involving the covalen immobilization of a monoclonal capture antibody on tosyl-activated MMPs, and the establishment of a sandwich-type immunoassay with a detector antibody coupled to a secondary labeled with AP. The sandwich magnetic immunoconjugates were captured on the surface of a screen-printed gold electrode (SPAuE) by means of a small magnet and, after addition of 4-aminophenyl phosphate as the AP substrate (Figure 1), square-wave voltammetry (SWV) was used for electrochemical detection. This MMPs-based immunosensor allowed a linear interval ranging between 0.01 and 100 ng·mL^−1^, a LOD of 0.005 ng·mL^−1^, and was applied to the determination of hGH in spiked human serum.

A disposable voltammetric immunosensor for early diagnosis of soybean rust through the determination of the fungus *Phakopsora pachyrhizi* was reported by Mendes et al. [17]. This approach, involving the use of Protein G (ProtG)-MMPs, a detector antibody labeled with AP, and DPV detection using α-naphtyl-phosphate as substrate, allowed achieving a linear concentration range from 5.0 to 45.0 μg·mL^−1^ and a LOD of 18 ng·mL^−1^.

Gamella et al. [18] developed and compared the performance of two different disposable amperometric immunosensors for D-dimer. This is a relevant marker for coagulation whose levels appear elevated in diverse clinical conditions. Indirect competitive and sandwich formats were designed based on the use of HOOC-MMPs and SPCEs. Both configurations exhibited linear calibrations within concentration ranges of clinical usefulness (0.084–1.9 and 0.06–1.0 μg·mL^−1^ for the indirect competitive and sandwich formats, respectively) and the corresponding LODs were 0.028 and 0.020 μg·mL^−1^, which are quite below the clinical threshold (0.5 μg·mL^−1^ D-dimer). The sandwich configuration was applied with excellent results to the analysis of undiluted spiked serum and two sera samples with a certified content of D-dimer.

The preparation and characteristics of a disposable amperometric magnetoimmunosensor for the specific detection and quantification of Staphylococcal protein A (ProtA) and *Staphylococcus aureus*, based on the use of ProtA-MMPs, TTF-modified Au-SPEs, and the establishment of a direct competitive assay involving HRP-labeled ProtA antigen, were reported [19]. This methodology provided a good selectivity against the most common foodborne pathogens originating from milk, and very low LODs (3.9 × 10^−9^ μg·mL^−1^ of ProtA and 1 cfu *Staphylococcus aureus* mL^−1^ in raw milk samples), and was successfully applied to the analysis of inoculated milk samples.

Conzuelo et al. [20] reported the preparation and performance of a disposable amperometric magnetoimmunosensor for the specific detection and quantification of tetracyclines (TCs) residues in milk. This strategy involved the use of ProtG-MMPs for immobilization of a selective capture antibody, SPCEs, and a direct competitive assay involving a tracer with HRP. Taking tetracycline (TC) as model antibiotic, the dynamic range extended between 12.5 and 676.2 ng·mL^−1^, and the calculated LOD value was 3.9 ng·mL^−1^. A good selectivity against other antibiotic residues that could be present in milk and dairy products was found. Noticeably, very low LODs (in the low ppb level) were achieved in untreated milk samples for four tetracycline antibiotics tested, TC, oxytetracycline (OTC), chlortetracycline (CTC) and doxycycline (DXC). Good results were obtained in the application of the magneto-immunosensor to the analysis of UHT whole milk spiked with TC, and a reference milk containing a certified content of OTC, after just a dilution with the working buffer.

Torrente-Rodríguez et al. [21] explored for the first time the utilization of a Strep-MMPs based immunoassay combined with the electrochemical detection of lactoperoxidase (LPO) activity for the determination of this enzyme. Strep-MMPs were used to immobilize biotinylated anti-LPO antibodies, and a direct-type immunoassay was carried out using amperometric detection and the H_2_O_2_/HQ system by detecting the enzymatic activity of the captured target enzyme. This label-free MMPs-based immunosensor provided a linear calibration plot in the 0.42–12.5 mg·mL^−1^ LPO range, and a LOD of 0.12 mg·mL^−1^. Moreover, reliable results were obtained in the rapid determination of the endogenous content of enzyme in different cow milk samples.

A single-step, separation and wash-free amperometric magneto-immunosensor using strips of eight graphite SPEs was prepared, and tested with the immunological interaction between an IgG (previously immobilized on tosyl activated-MMPs) and its specific antibody (anti-IgG-HRP) model system [22]. Two different approaches to perform the amperometric detection were compared: one was based on the use of glucose oxidase (GOD) and Prussian Blue (PB)-modified SPEs, and the second one used pristine SPEs. Results using hydroquinone (HQ) as redox mediator demonstrated that the approach using pristine SPEs proved to be easier and more practical and resulted in a wider dynamic range for detection of anti-rabbit IgG-HRP concentrations.

Taleat el al. [23] developed a method for the ultrasensitive detection of human MUC1 cancer biomarker based on an electrochemical sandwich-type immunoassay using MMPs functionalized with ProtG and graphite SPEs. A pair of primary (immobilized on ProtG-MMPs) and secondary antibodies was used to sandwich the MUC1 protein, and a third antibody conjugated with HRP, for labeling. The determination was carried out by DPV upon H_2_O_2_ addition in the presence of acetaminophen acting as the redox mediator. A linear relationship between peak current and human MUC1 concentration was obtained over the 0–25 ng·mL^−1^ range, with a LOD of 1.34 ng·mL^−1^. This immunosensor was applied to the determination of the target biomarker in 200 times diluted human serum from breast and ovarian cancer patients.

An electrochemical magnetoimmunosensor was also developed for the detection of anti-transglutaminase antibodies (ATG2) in celiac disease [24]. The approach involved the selective capture of ATG2 onto transglutaminase enzyme (TG2) covalently immobilized on tosyl activated-MMPs and their labeling using HRP-conjugated secondary antibodies. The electrochemical responses obtained by SWV at SPCEs using the system o-phenylenediamine/H_2_O_2_ were correlated with the ATG2 concentration. Using a commercial available standard antibody solution (whole serum), the methodology provided a dynamic range from 1:10,300 to 1:3000 and a LOD of 1:14,200. This electrochemical immunosensor was applied to analyze 48 human sera samples (29 from celiac disease patients and 19 from healthy individuals). Analysis of the results by receiver-operating characteristic plot (ROC) evidenced that a cut-off value of 16.95 units provided respective percentages of specificity and sensitivity of 84 and 100.

Two different types of MMPs (ProtA- or Strep-functionalized) were analytically compared for the development of electrochemical immunosensors for ceruloplasmin (Cp), a relevant inflammation biomarker, using direct competitive formats with synthesized AP-Cp conjugate [25]. The determination of Cp was made by DPV measurement of 1-naphthol generated after addition of 1-naphthyl phosphate upon capturing the corresponding MMPs immunoconjugates onto SPCEs. Linear ranges of calibration plots and LODs were respectively 0.1–1000 μg·mL^−1^ and 0.040 μg·mL^−1^ (Prot A-MMPs), and 0.025–20 μg·mL^−1^ and 0.018 μg·mL^−1^ (Strept-MMPs). The Strept-MMPs-based immunosensor was applied with satisfactory results to the determination of Cp in spiked human serum.

Esteban-Fernández de Ávila et al. developed highly sensitive MMPs-based immunosensing strategies for human C-reactive protein (CRP) [26] and human cardiac troponin T (cTnT) [27] determination using sandwich formats onto HOOC-MMPs and Strep-MMPs, respectively, as well as immunoreagents labeled with HRP and amperometric detection involving H_2_O_2_/TMB at Au-SPEs. A wide range of linearity (0.07–1000 ng·mL^−1^) was exhibited by the CRP magnetoimmunosensor, with a LOD value (0.021 ng·mL^−1^) far below the clinical cut-off (1000 ng·mL^−1^) corresponding to the severity of risk for cardiovascular disease. This device was successfully applied to the determination of CRP in an international CRP serum standard. Regarding cTnT, the developed methodology achieved a LOD value of 0.017 ng·mL^−1^ and was also evaluated by application to serum samples with good results.

Same authors also developed an amperometric magnetoimmunosensor involving an indirect competitive format for detection of the amino-terminal pro-B-type natriuretic peptide (NT-proBNP) [28]. The antigen was covalently immobilized onto HOOC-MMPs and further incubated in a mixture solution containing the antigen at variable concentrations, and a fixed concentration of the HRP-labeled antibody detector. The amperometric detection was performed after magnetic capturing of the immunoconjugate-bearing MBs at Au-SPEs and using the system H_2_O_2_/TMB. The analytical characteristics of the developed method using this magnetoimmunosensor in 10-times diluted human serum samples, were especially attractive. Thus, a linear range (0.12–42.9 ng·mL^−1^) and a LOD of 0.02 ng·mL^−1^, useful in clinical diagnosis of chronic heart failure in the elderly, as well as for classifying patients at risk of death after heart transplantation, were achieved. Moreover, as in other cases, this immunosensor was successfully applied to the analysis of spiked human serum.

Human interleukin-6 (IL-6) is a cytokine playing a prominent role in the inflammatory response. A magnetoimmunosensor design for this protein involving covalent immobilization of anti-IL-6 antibodies onto HOOC-functionalized MMPs and the performance of a sandwich-type immunoassay with signal amplification using poly-HRP-streptavidin conjugates was also developed [29]. By amperometric detection involving the H_2_O_2_/HQ system at SPCEs, a linear calibration plot in the 1.75 to 500 pg·mL^−1^ concentration range, and a LOD of 0.39 pg·mL^−1^ IL-6 were obtained. This immunosensor was validated by analysis of spiked urine and saliva samples providing results that statistically agreed with those obtained with a commercial ELISA kit but in a much shorter time.

Two different amperometric immunosensors were described by Campuzano et al. [30] for fibrinogen determination based on a novel specific nanobody expressed in *Escherichia coli*, and MMPs. Direct and indirect competitive magnetoimmunosensing configurations using His-Tag-Isolation-MMPs and COOH-MMPs for the immobilization of the recombinant nanobody or the antigen, respectively, were tested and compared. In the direct competitive format, fibrinogen and biotinylated-fibrinogen competed for the binding sites of the immobilized nanobody binding sites. Furthermore, the indirect approach involved competition between free fibrinogen in solution and immobilized fibrinogen for binding to the specific biotinylated nanobody at a fixed amount. In both cases, the captured biotinylated antigen or nanobody was labeling with Strep-HRP, and detection was accomplished by amperometry at SPCEs with the system H_2_O_2_/HQ. Although the indirect competitive immunoassay allowed a better analytical performance and a LOD of 0.044 μg·mL^−1^, the analytical utility of both approaches was proved by application to an international standard for fibrinogen plasma. Moreover, the same authors developed also other amperometric bioplatform for fibrinogen based on an indirect competitive format with an HRP-labeled commercial antibody and biotinylated fibrinogen immobilized onto Strep-MMPs [31]. Using also amperometric detection involving H_2_O_2_/HQ at SPCEs, this methodology demonstrated an excellent analytical behavior showing a dynamic range between 0.004 and 0.8 μg·mL^−1^ and providing a LOD value of 0.8 ng·mL^−1^. This magnetoimmunosensor was used to determine fibrinogen in a commercial plasma sample with certified content, taking a total assay time of 80 min, after a simple sample treatment consisted just of a dilution in the buffer assay.

An electrochemical immunosensor for the determination of tumor necrosis factor alpha (TNFα) was also reported [32]. Sandwich immunocomplexes were magnetically captured on the surface of working SPCEs and, upon addition of HQ and H_2_O_2_, the amperometric responses were measured. This MMPs-based bioscaffold provided LODs of 2.0 (36 fM) for standard solutions, and 5.8 pg·mL^−1^ (105 fM) for spiked human serum, these values lying well in the range of clinical relevance. Using a similar approach, same authors also developed other amperometric magnetoimmunosensor for the breast cancer biomarker ErbB2 [33]. The method exhibited a very low LOD value (26 pg·mL^−1^) which far below the cut-off established for this biomarker in serum (15 ng·mL^−1^). Moreover, good results were obtained by application of the immunosensor to the determination of the target protein in human serum and cell lysates in the absence of matrix effect. In addition, the developed bioplatform made possible the direct assessment of ErbB2 status in intact breast cancer cells, thus demonstrating that the new magnetoimmunosensing bioscaffold constitutes a useful and truthful analytical tool in the diagnosis of breast cancer by either ErbB2 protein determination in serum or detection of breast cancer cell status.

An electrochemical immunosensor for ghrelin (GHRL) determination was also developed by using a direct competitive format involving GHRL and biotinylated-GHRL, Strep-AP as enzymatic tracer and ProtG-MMPs as solid support [34]. By measuring the DPV signal of 1-naphtol produced upon 1-naphtyl phosphate addition at SPCEs, a calibration plot with a linear range between 10^−3^ and 10^3^ ng·mL^−1^ and a LOD value of 7 pg·mL^−1^ (much smaller than those provided by commercial ELISAs) were achieved. Results presented demonstrated the usefulness of this immunosensor by analyzing spiked human saliva samples.

Jodrá et al. [35] described an amperometric magnetoimmunosensor for Ochratoxin A (OTA). The implemented strategy was founded on the direct competitive assay between OTA and an HRP-labeled derivative on selective capture antibody immobilized on ProtG-MMPs, as well as the amperometric transduction at SPCEs using the system H_2_O_2_/HQ. The method achieved a dynamic concentration range between 1.3 and 153.8 μg·L^−1^ and a LOD of 0.32 μg·L^−1^, well below the legislative requirements of this mycotoxin in soluble coffee samples (10 μg·kg^−1^). These authors developed a simultaneous simplified calibration and coffee analysis strategy which provided recoveries of 90% in spiked coffees.

This same group developed also a very similar MMPs-based immunosensing strategy for the detection of fumonisins B1 (FB1), B2 (FB2) and B3 (FB3) [36]. Direct competitive formats and ProtG-MMPs as solid support were utilized to perform the immunoreactions using FB1 as model fumonisin. Amperometric detection at SPCE with the system H_2_O_2_/HQ, allowed estimated LOD value and dynamic range of 0.33 μg·L^−1^ and 0.73–11.2 μg·L^−1^, respectively. The reliability and accuracy of this strategy was investigated by application to the analysis of reference material of certified maize, and 10-fold diluted spiked beer samples, getting recovery dates of 85%–96%.

Lipoprotein (a) (Lp(a)) biomarker is a relevant predictor of risk for cardiovascular disease. Kaçar et al. [37] described a very sensitive electrochemical magnetoimmunosensor for rapid detection of Lp(a) in human serum using a sandwich configuration involving HOOC-MMPs, together with a selective capture antibody, a biotin-labeled detector antibody, and a Strep-HRP conjugate. The resulting MMPs bearing the sandwiched immunoconjugates were captured on the surface of a SPCE working electrode, and the extent of the affinity reaction was monitored amperometrically using the H_2_O_2_/HQ system. The method exhibited a wide linear response range (0.01–0.5 μg·mL^−1^) and a LOD of 4 ng·mL^−1^. This value was significantly below the minimum cut-off value of 300 mg·L^−1^ established in serum to prognosticate the probability of cardiovascular risk, and demonstrated the utility for the determination of Lp(a) in a reference serum containing a certified quantity of protein.

A label-free ProtG-MMPs-based immunosensing approach was developed for the determination of acetaminophen (APAP) [38]. APAP, selectively captured onto ProtG-MMPs modified with a specific antibody, was detected by DPV at SPCEs. The developed approach, which allowed obtaining a LOD of 1.76 μM and a linear concentration range between 5.28 μM and 0.75 mM, was utilized for the determination of APAP in two pharmaceutical products.

Pt-SPEs and HOOC-MMPs were combined by Čadková [39] to design a voltammetric immunosensor for monitoring the serious food allergen ovalbumin (OVA). Using a sandwich format and a detector antibody conjugated with HRP, the electrochemical signal was monitored by LSV (linear sweep voltammetry) using the system H_2_O_2_/thionine. This newly established method exhibited high sensitivity and demonstrated its suitability for OVA quantification in the concentration range of 11 to 222 nM providing a LOD of 5 nM.

Novel magnetoimmunosensing platforms were developed for the sensitive and selective determination of Ara h 1 [40] and Ara h 2 [41], two peanut allergenic proteins. These platforms are based on sandwich formats with HRP-labeled immunoreagents onto HOOC-MMPs and amperometric detection (H_2_O_2_/HQ) at SPCEs. The developed immunosensors exhibited calibrations with wide ranges of linearity (20.8–1000.0 ng·mL^−1^ for Ara h 1 and 87–10,000 pg·mL^−1^ for Ara h 2) and low LODs with respective values of 6.3 ng·mL^−1^ and 26 pg·mL^−1^. The analytical utility of these approaches was demonstrated by the accurate determination of content of the endogenous target protein in various food extracts. Moreover, results presented demonstrated the feasibility of these platforms to detect the peanut presence in undiluted saliva samples [40] as well as traces of the peanut allergen (0.0005% or 5.0 mg·kg^−1^) in spiked wheat flour [41].

Same authors designed also interesting immunoassay platforms based on MMPs for the detection of β-lactoglobulin (β-LG) [42] and α-lactalbumin (α-LA) [43], two of the main milk allergenic proteins. Both immunosensing platforms, using also sandwich formats with HRP-labeled immunoreagents onto HOOC-MMPs and amperometric detection at SPCEs, provided excellent analytical performances with wide linear ranges (2.8–100 ng·mL^−1^ for β-LG and 37.0–5000 pg·mL^−1^ for α-LA) and low LODs (0.8 ng·mL^−1^ for β-LG and 11.0 pg·mL^−1^ for α-LA). The immunosensors were successfully applied for the detection of the target proteins in different types of milk with no matrix effect after just sample dilution, providing results in good agreement with those given by commercial ELISA methods.

Vidal et al. [9] recently reported a multiple electrochemical immunosensor based on a competitive assay for fast determination of unmetabolized cocaine in urine, saliva and human serum. The immunosensor consisted of an array of eight SPCEs, and used immunoconjugates of anti-cocaine polyclonal antibodies onto ProtG-MMPs, as well as direct competitive formats between the analyte and HRP-labeled analyte. The amperometric detection was performed using the H_2_O_2_/HQ system. This multi-electrochemical competitive immunosensor allowed LODs of 0.09 (PBS), 0.36 (urine), 0.09 (saliva), and 0.63 (human serum) ng·mL^−1^ of cocaine.

The determination of estrogen receptor α (ERα) protein, a relevant breast cancer hormonal receptor, was the objective of an electrochemical magnetoimmunosensor developed by Eletxigerra et al. [44]. Specifically functionalized HOOC-MMPs with sandwich immunocomplexes comprising HRP immunoreagents, and amperometric detection at SPCEs using the H_2_O_2_/HQ system, resulted in highly selective and sensitive ERα detection with a LOD value of 19 pg·mL^−1^. The demonstrated capabilities of this magnetoimmunosensing platform for ERα quantitation in spiked human serum and cell lysates with no matrix effect, as well to assess ERα in intact breast cancer cells, make it competitive in terms of simplicity, rapidity and reliability, with conventional strategies applied in clinical practice for diagnosis, monitoring and follow-up of metastatic breast cancer.

Very recently, an amperometric MMPs-based immunosensing strategy was developed for miR-205 determination. This approach involved the use of ProtG-MMPs modified with the antibody AbS9.6 specific for the DNA/RNA heteroduplexes as selective microcarriers to capture the hybrids previously formed in solution by homogeneous hybridization between the biotinylated complementary DNA probe and the target miRNA [45]. This immunosensing approach exhibited a dynamic range from 8.2 to 250 pM and a LOD of 2.4 pM (60 amol) of the synthetic target miRNA. The usefulness of the method was validated by analysis of total RNA (RNAt) extracted from human tumor tissues and cancer cell lines, which fully demonstrated its potential to carry out the determination of mature miRNAs in this type of complex samples.

A rapid disposable magneto-actuated immunosensor was also developed for determination pf endoglin, a relevant biomarker in cancer and rheumatoid arthritis, in serum samples [46]. A specific antibody was immobilized onto HOOC-MBs to selectively capture the target protein. Then, the conjugate was sandwiched with a secondary HRP-labeled antibody, and the immunocomplexes attached to the MMPs were amperometrically detected at SPCEs using the HQ/H_2_O_2_ system. The immunosensing platform could detect 5 pmoles of endoglin in 25 μL of sample (0.2 ng·mL^−1^) in 30 min providing results statistically similar to those given by a commercial ELISA kit for the determination of endogenous endoglin in human serum.

### 2.2. Magnetic Microparticles-Screen Printed Electrodes Electrochemical DNA/RNA Biosensors

MMPs benefits have been also coupled with those of SPEs for the preparation of DNA (or RNA) electrochemical biosensors. Some illustrative examples are commented below.

A disposable amperometric MMPs-based DNA sensor coupled to asymmetric PCR (aPCR) was developed for the ultrasensitive determination of *Streptococcus pneumoniae* [47]. This approach was based on the selective hybridization of specific biotynilated capture DNA probe, complementary to a specific region of the pneumococcal *lytA* gene. Strep-MMPs were modified with the biotinylated synthetic target or the predominantly 235-base single-stranded (ss) amplicon generated by direct aPCR (daPCR) from bacterial cultures. In a final step, the biotinylated hybrid attached to the Strep-MMPs was labeled with Strep-HRP, and determination was performed by amperometric detection using H_2_O_2_ at TTF-Au-SPEs. While a LOD value of 5.1 nM for a 20-mer synthetic target DNA was obtained without any amplification protocol, a LOD of 1.1 nM was estimated for the ss-aPCR amplicon. Results demonstrated that daPCR products could be prepared with as few as 2 cfus of *S. pneumoniae*. Furthermore, by application of this methodology, no cross-reaction with *Streptococcus mitis* (a closely related streptococcus) was observed, and data also demonstrated the possibility of discrimination between samples of non-inoculated blood and urine and samples inoculated with *S. pneumoniae* at a very low concentration (10^3^ CFU·mL^−1^). Indeed, this approach was successfully validated with 109 clinical samples of diverse origins providing both sensitivity and specificity of around 90% [48].

Erdem et al. [49] developed a sensitive and selective enzyme-linked sensor technology for electrochemical detection of microRNAs (miRNAs) using a multi-channel array of 16 screen-printed carbon electrodes (MUX-SPCE16s) and Strep-MMPs. Strep-MMPs modified with specific biotinylated DNA capture probes were used for selective capture of the biotinylated miRNA target and, after labeling with Strep-AP the resultant biotinylated hybrid, the oxidation signal of α-naphthol generated by hydrolysis of α-naphthylphosphate in the presence of AP was measured by LSV at the MUX-SPCE16s. It is important to mention than in this case only the supernatant of the hydrolysis reaction and not the modified MMPs was transferred to the MUX-SPCE16s to perform the LSV detection. Using miRNA-15a as model target, the developed methodology provided a calibration plot with a linear interval ranging between 2.5 and 10.0 μg·mL^−1^, achieving a LOD of 0.114 μg·mL^−1^ (34.20 fmole in 3 μL sample).

**Figure 2:**
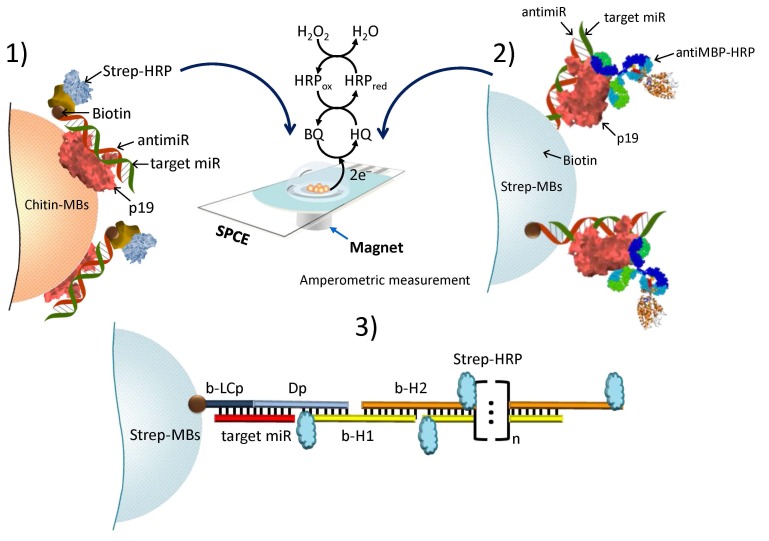
Strategies developed by Pingarrón´s group for miRNA determination based on the coupling of micro-magnetic particles (MMPs) and amperometric detection at screen-printed carbon electrodes (SPCEs) using p19 protein as capture bioreceptor (**1**) [50] as detector bioreceptor (**2**) [51]; and a sandwich hybridization coupled to an HCR amplification strategy (**3**) [52]. Adapted from Refs. [50,51,52] with permission.

Pingarrón´s group has developed recently different attractive strategies for miRNAs detection involving MMPs and amperometric detection using the system H_2_O_2_/HQ at SPCEs. Two of them are based on the employment of the viral protein p19 as specific bioreceptor for the selective capture (previously immobilized at Chitin-MMPs) [50] or detection [51] of duplexes of RNA formed by target miRNA and a specific biotinylated RNA probe hybridization in homogeneous solution or at the surface of Strep-MMPs. The commercial p19 used as receptor was cloned and expressed in *Escherichia coli* as a fusion protein with an amino terminal maltose binding protein (MBP) and a carboxy terminal chitin binding domain (CBD). Using Strep-HRP or an antiMBP-HRP for labeling, these approaches allowed LODs of 0.4 and 4.2 fmol of the synthetic miR-21 target. More recently, a novel approach involving a sandwich hybridization assay onto Strep-MMPs and hybridization chain reaction (HCR) amplification has been developed [52]. The target miRNA-21 contains 11-base complementary sequences to both the biotinylated capture probe (b-LCp) and the detector probe (Dp) (Figure 2). This latter acts also as the initiator strand of an HCR amplification in the presence of two biotinylated hairpin sequences leading to the formation of a long nicked double-helix structure bearing a large number of biotin molecules used to capture multiple HRP enzymes in a highly ordered way thus allowing an efficient amplification of the final amperometric signal. This HCR-sandwich based approach allowed a linear increase in the amperometric signal from 0.2 to 5.0 nM and a LOD value of 0.06 nM (5.0 fmol in 25 μL sample). The applicability of these three different approaches has been demonstrated for the determination of the mature miRNAs target directly in raw RNA_t_ extracted from human tissues and cancer cells. It is worth mentioning that none of these approaches for miRNA determination requires previous reverse transcription into cDNA, which implies a lower cost and a shorter analysis time.

**Figure 3:**
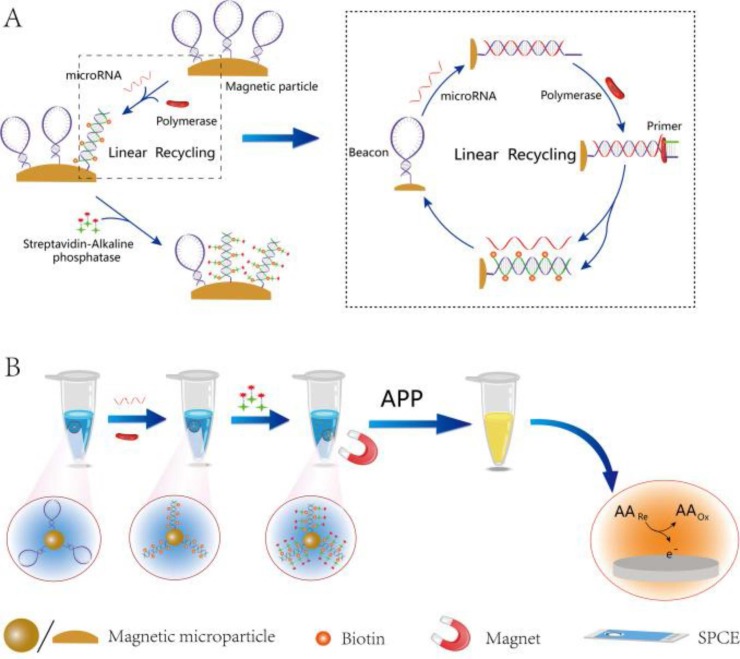
Representative diagram for the isothermal strand-displacement polymerase reaction (ISDPR) amplification (**A**) and the fabrication process of the multienzyme functionalized MMPs and oxidation of ascorbic acid (AA) at an SPCE (**B**). Reprinted from Ref. [4] with permission.

A MMPs-assisted multienzyme-functionalized isothermal strand-displacement polymerase reaction (ISDPR) was developed by Ma et al. [4] for quantification of miRNAs. Recognition of aminated molecular beacons attached to HOOC-MMPs by target miRNA triggered a phi29-mediated ISDPR, which can produce biotin-modified sequences on the MMCs labeled with Strept-AP in a final step (Figure 3). The ascorbic acid (AA) generated by AP using 2-phospho-L-ascorbic acid (AAP) as substrate was detected by DPV upon placing the supernatant of enzymatic reaction at SPCE. This method provided a linear range extending between 10 fM and 10 nM and a LOD value of 9 fM for the target miRNA (miRNA-21), and also it was successfully applied to the determination of miRNAs in breast cancer tissues.

### 2.3. Other Magnetic Microparticles-Screen Printed Electrodes Electrochemical Affinity Approaches

Gamella et al. [53] developed an amperometric affinity sensor based on His-Tag-Isolation-MMPs for the 30-min determination of β-lactam antibiotics in milk using the functionalized microparticles to immobilize a recombinant Histidine-tagged penicillin-binding protein (His-PBP). A direct competitive assay was performed involving a tracer with HRP and amperometric detection at SPCEs using the H_2_O_2_/HQ system. This methodology provided LOD values in the low parts-per-billion level in untreated milk samples for the six antibiotics tested, and was able to detect in a selective way exclusively the active form of β-lactam antibiotics showing high affinities for both cephalosporins and penicillins.

An original magneto-immuno-PCR electrochemical approach was also reported for direct and highly sensitive detection of *Streptococcus pneumoniae* [54]. In order to amplify a characteristic 235-bp region of the gene coding for the major pneumococcal autolysin (*lytA*), the developed methodology involved the use of direct asymmetric PCR (DaPCR) of the bacteria attached to capture antibody-ProtA-MMPs. Subsequently, hybridization of biotinylated amplicons and the predominantly single-stranded generated was performed onto Strep-MMPs modified with a specific biotinylated 20-mer capture probe. After labeling the resultant biotinylated hybrid with Strep-HRP, amperometric detection was performed using H_2_O_2_ at TTF-modified SPAuEs upon magnetic capture of the modified MMPs. Using this methodology, calibration plots were constructed for R6 and Dawn serotypes, and the LOD values were of 132 and 130 cfu·mL^−1^, respectively, these being about 100–1000 times lower than those provided by using this same methodology without PCR amplification [13].

### 2.4. Multiplexing Using Magnetic Microparticles-Screen Printed Electrodes

SPEs technology is particularly well suited for multiplexing purposes with commercial availability of several low cost electrochemical platforms that can be considered as an attractive alternative for point-of-care analysis.

Erdem et al. [55] described a label-free voltammetric method for simultaneous determination of three different microRNAs deregulated in Alzheimer disease (miRNA-16, miRNA-15a and miRNA-660). Strep-MMPs modified with biotinylated inosine substituted DNA probes were used for selective capture of the target miRNAs and, after passive adsorption of the hybrid released from the MMPs onto the working electrodes of the MUX-SPCE16s, the oxidation response of guanine was measured using DPV. With miRNA-16 as the model, a LOD of 4.3 pmole in 3 μL sample was estimated and the guanine response gradually increased till 80 μg·mL^−1^.

Amino-terminal pro-B-type natriuretic peptide (NT-proBNP) and C-reactive protein (CRP) are two very promising biomarkers for cardiac risk prediction whose clinically relevant cut-off concentrations differ by three orders of magnitude. For their simultaneous determination in human serum, a rapid magnetoimmunosensor was developed [56]. HOOC-MMPs were used to covalently immobilize a specific capture antibody or the target antigen, and the quantification of CRP and NT-proBNP was performed by using sandwich and indirect competitive configurations, respectively, as well as HRP-labeled tracers and amperometric detection (H_2_O_2_/TMB) at SPdCEs. The developed methodology showed linear and dynamic ranges of 2.0–100 ng·mL^−1^ and 2.5–504 ng·mL^−1^ respectively for CRP and NT-proBNP, and LODs of 0.47 ng·mL^−1^ for both biomarkers, and it was validated by application to a CRP serum international standard also spiked with NT-proBNP.

**Figure 4:**
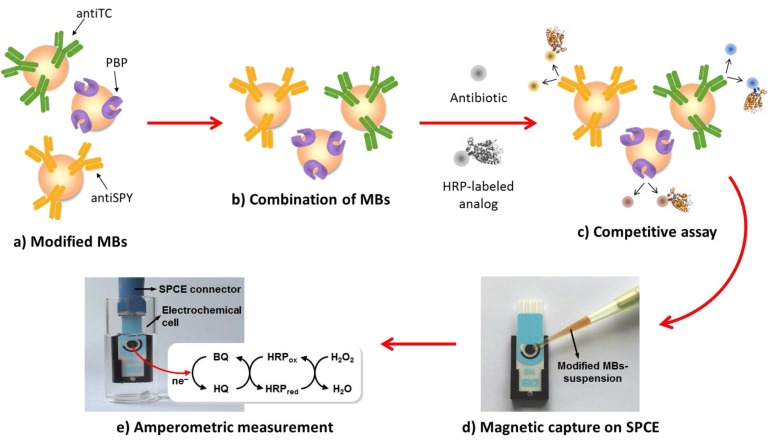
Schematic display of the multi-antibiotics magnetosensor developed. The modified MMPs (MBs in the Figure) (**a**) are commingled together (**b**) and incubated with the sample in the presence of a fixed amount of the three enzyme-labeled analogs, thus establishing a direct competitive assay (**c**), the MBs are then captured on the surface of a SPCE inserted on the magnet holding block (**d**) and SPCE-magnet holding block immersed in the electrochemical cell (**e**). Reprinted from Ref. [57] with permission.

An interesting strategy for the rapid multiplexed screening of antibiotic residues in milk, applied to cephalosporins (CPHs), sulfonamides (SAs) and TCs, was reported by Conzuelo et al. [57]. This involved the use of SPCEs, a mixture of MMPs modified with 3-target specific, and the establishment of direct competitive assays with HRP-labeled tracers (Figure 4). Specific antibodies immobilized onto ProtG-MMPs and His_6_-tagged penicillin-binding protein (PBP) immobilized onto His-tag isolation-MMPs were used as selective capture microcarriers for SAs, TCs and CPHs, respectively. The amperometric responses measured at SPCEs using the H_2_O_2_/HQ system allowed to evaluate the extent of affinity reactions. Noticeably, this methodology makes possible discrimination between raw milk and uncontaminated UHT samples and samples containing antibiotic residues at the maximum residue limits (MRLs) in only 5 min, by applying a simple and short pretreatment.

A method for quantification of genetically modified organisms (GMO) was also developed by Manzanares-Palenzuela et al. [58]. Round-Up Ready Soybean (RRS) was selected as a model GMO. This is an herbicide-resistant form of soybean that was genetically engineered representing one of the most successful achievements of crop biotechnology. Thus, in 2013, it was extending as more than 75% of worldwide soybean plantations. Two DNA sequences, a fragment of the endogenous lectin (Lec) gen for soybean and an event-specific sequence from RRS, were targeted via sandwich hybridization onto specific biotinylated DNA probes-modified Strep-MMPs and detection probes labeled with digoxigenin (Dig) or fluorescein isothiocyanate (FITC). Dual enzymatic labeling using Fab fragments of anti-FITC and anti-Dig conjugated to HRP or AP, respectively was also performed. Electrochemical measurements of enzymes activity were made in parallel at individual SPCEs using chronoamperometry (HRP, H_2_O_2_/TMB system) and DPV (AP, 1-naphthyl phosphate substrate). The affinity assay provided a linear range of 2–250 pM for both targets, and LOD values of 650 and 190 fM, respectively, for the event and the taxon-specific targets. These results demonstrated the applicability of this method for the determination of GMO at levels below the European labeling threshold (0.9%) in foods.

Based on the methodology previously developed by the same group for the determination of a single miRNA [50], Torrente-Rodríguez et al. [59] implemented the first affinity sensor able to simultaneously detect in one single experiment, two different miRNAs expression. The approach implied preparation of two distinct batches of p19-protein modified MBs, one for each target miRNA, and performing the amperometric detection at dual SPCEs. This scaffold exhibited sensitive and selective detection of miRNA-21 and miRNA-205 with linear ranges from 2.0 to 10.0 nM and LODs of 0.6 nM (6 fmol) for both miRs without need for amplification, in a short time (less than 2 h). The utility of the approach was demonstrated by determining the endogenous levels of both targets in total RNA extracted from human breast cancer tissues and cell lines.

An amperometric magnetoimmunosensing platform was developed by Eletxigerra et al. [60] for the simultaneous detection of estrogen α (ERα) and progesterone (PR) breast-cancer related receptors. The fundamentals of this dual immunosensing platform design relied on the use of two different batches of functionalized MMPs bearing HRP-labeled sandwich immunocomplexes specific for each protein receptor and magnetic capturing on the corresponding of a dual SPCE. Amperometric detection of H_2_O_2_ in the presence of HQ as redox mediator was used to determine each receptor concentration. This approach provided linear ranges of 73–1500 (PR) and 63–2000 pg·mL^−1^ (ERα), and LODs of 22 (PR) and 19 pg·mL^−1^ (ERα). This dual platform demonstrated its applicability as it could discriminate raw lysates of MCF-7 and MDA-MB-436, two types of breast cancer cells with significantly different PR/ERα expression levels.

Torrente-Rodríguez et al. [61] developed other MMPs-based dual amperometric disposable platform for the direct detection in saliva of IL-8 protein and IL-8 mRNA, two relevant oral cancer biomarkers. The methodology involved the use of two types of MMPs (Strept-MMPs and HOOC-MMPs), and specific antibodies against IL-8 protein. A specific biotinylated hairpin DNA sequence for IL-8 mRNA, labeling with HRP in both cases, and amperometric detection at SPdCEs were also used. LOD values of 0.21 nM for IL-8 mRNA and 72.4 pg·mL^−1^ for IL-8 protein were found in undiluted saliva samples. It is worth mentioning that the latter LOD is 23 times lower than the clinical cut-off value (600 pg·mL^−1^) in saliva established to discriminate healthy individuals from oral cancer patients, as well to highlight the absence of statistical differences between the calibration plots constructed with saliva from ten different healthy individuals, this allowing the possibility of analyze all saliva samples using a single calibration plot. Furthermore, the dual electrochemical biosensor was successfully used for the direct analysis of spiked raw saliva, where the both biomarkers were determined, and also to analyze saliva samples from seven healthy individuals for detecting the endogenous content of IL-8 protein. Providing results were in good agreement with those obtained by conventional ELISA methodology.

Very recently, a novel MMPs-based immunosensing approach was also described for the rapid and simultaneous determination of Ara h 1 and Ara h 2, the main peanut allergenic proteins [62]. It involves sandwich-type immunoassays onto HOOC-MMPs and amperometric detection (H_2_O_2_/HQ) at SPdCES. This methodology exhibited LOD values of 18.0 and 0.07 ng·mL^−1^, respectively, for Ara h 1 and Ara h 2, requiring an assay time as short as 2 h. The utility of the reported approach was investigated by analyzing various food extracts to determine the endogenous concentration of both allergens, as well as wheat flour spiked samples to detect trace amounts of peanut allergen (0.0001% or 1.0 mg/kg). The developed platform provided better LODs than those of commercial allergen specific ELISA kits.

## 3. Magnetic Nanoparticles

Properties of magnetic nanobeads (φ ≤ 100 nm) in terms of structure, biological reactivity or biorecognition strategies, are similar to those from their microsized counterparts. However, their higher surface/volume ratio provides much more binding sites for biomolecules anchoring, these often giving better S/N ratios [63]. Moreover, the use of nanomaterials avoids the size mismatch existing between magnetic microbeads and nanometric biological reagents, also providing biocompatibility. Furthermore, due to their unique properties, magnetic nanoparticles (MNPs) make possible faster electron transfer between redox systems and the electrode surface, and can act as electrocatalysts for molecules such as hydrogen peroxide, implied in biochemical reactions of interest [64]. In addition, MNPs are used not only for the preparation of bioconjugates on the electrode surface but also as labels for signal amplification.

Due to their biodegradability and high biocompatibility, magnetite (Fe_3_O_4_) and maghemite (γ-Fe_2_O_3_), are the main representative materials of MNPs used for biological applications [65]. Diverse physical, chemical, and microbial methods have been proposed for the synthesis of low cost, uniformly sized particles exhibiting large surface areas and diameters usually ranging beween units and various tens of nanometers [66]. Core-shell nanomagnetics and nanocomposites have also been used for electrochemical biosensing. Among them, core-shell Fe_3_O_4_@AuNPs and Fe_3_O_4_@SiO_2_ are the most common [67]. In the first case, the presence of gold nanoparticles assures the excellent conductivity and adsorption ability of magnetic material and, furthermore, SiO_2_ surface enhances the stability of nanoparticles and provides a good surface for bioreagents binding. Other oxides such as TiO_2_ have also been used to prepare core-shell magnetic nanoparticles with specific properties. Metal-doped iron oxides (MFe_2_O_4_, with M = Co or Mn, among others), with enhanced magnetic properties of the resulting spinel metal ferrites, have also been proposed. Moreover, it is currently becoming common the use of polymers for coating the surface of nanoparticles in order to increase the immobilization capacity, as well as the combination of magnetic nanoparticles with carbon nanomaterials such as carbon nanotubes or graphene to take advantage of properties of the resulting hybrids.

### 3.1. Magnetic Nanoparticles-Screen Printed Electrodes Electrochemical Immunosensors

The special properties of gold nanoparticles, particularly their high conductivity and the ability to stably adsorb biomolecules, make them the most used nanomaterial in combination with magnetic nanoparticles for the preparation of electrochemical affinity biosensors. One example is the use of core/shell Fe_3_O_4_@AuNPs conjugated with anti-*Salmonella* antibodies for the development of an electrochemical immunosensor for *S. typhimurium* involving labeling with CdS nanocrystals for the detection by stripping SWV. A thermal decomposition method by reaction of Fe(III) acetylacetonate with 1,2-hexadecanediol in the presence of both oleic acid and oleylamine was used to obtain magnetite nanoparticles [68], that were further coated by gold in toluene at 85 °C. The resulting MNPs were functionalized with a mixed monolayer of 2-mercaptoethanol and 12-mercaptododecanoic acid and used to covalently immobilize the capture antibody. Separately, CdS nanocrystals were used to immobilize also anti*-Salmonella* antibodies, and a sandwich immunoassay involving SPCE/Fe_3_O_4_@AuNPs-Ab and CdS-Ab was performed. The anodic stripping current from cadmium registered by SWV provided a sigmoidal calibration plot between 10 and 10^6^ cells·mL^−1^, with a LOD value of 13 cells·mL^−1^ for an incubation time of 20 min. The method was used for analyzing spiked milk samples [69]. It should be noted that this method demonstrated the possibility of determine the bacteria in milk at low concentrations and in less than 1 h.

Gan et al. [70] prepared an electrochemical immunosensor for carcinoembryonic antigen (CEA) using Fe_3_O_4_@AuNPs to assemble HRP-anti-CEA and BSA. The magnetoconjugate was immobilized onto a SPCE modified with a carboxylated MWCNTs-thionine (Thi)-Nafion composite and, through one-step immunoassay format, the immunosensor was incubated with CEA. Immunoconjugation with the antigen produced a decrease in the catalytic efficiency of HRP for the oxidation by H_2_O_2_ of immobilized thionine. This provided a decrease in the electrochemical current which, under the optimized conditions, could be proportionally related to the concentration of the antigen between 0.1–5.0 and 5.0–80 ng·mL^−1^ with a LOD value of 0.03 ng·mL^−1^. With the aim of obtaining a label-free, simple, fast, reproducible and non-toxic immunosensor for tumor biomarkers, AuNPs and Fe_3_O_4_ were immobilized onto MWCNTs previously functionalized with redox-active hemin and the polyelectrolyte PDDA (poly(dimethyldiallylammonium)). Alpha fetoprotein (AFP) was used as the model antigen, whereas anti-AFP was adsorbed on the AuNPs surface, and the nanocomposite was incorporated to SPCE. In the presence of AFP antigen, the immunosensing event affected the electron transfer of hemin given a decrease of the DPV peak current, this providing a linear response for AFP between 0.1 and 200 ng·mL^−1^ with and LOD of 0.04 ng·mL^−1^ [71]. The developed immunosensor constitute a good example of a one-step immunoassay device providing good sensitivity and selectivity. However, it has only been applied to AFP standard solutions.

The same group developed a similar immunosensor for high-sensitivity (hs)-CRP using Fe_3_O_4_@AuNPs functionalized with 2-aminoethanethiol to immobilize HRP-anti-hs-CRP. SPCEs modified with iron phtalocyanine (FePc) and chitosan were used to magnetically attract HRP-anti-hs-CRP/Fe_3_O_4_@AuNPs conjugates, so that in this case, the immunoconjugation with the antigen inhibited the catalytic efficiency of HRP to the H_2_O_2_ reduction of FePc. This inhibition was proportional to the concentration of the antigen within 1.2 to 200 ng·mL^−1^ concentration range, and the LOD was 0.5 ng·mL^−1^ [72]. An important characteristic of the immunosensor is the reusability as it can be regenerated, this greatly decreases the cost of detection.

Clenbuterol (CLB) is a β-agonist that can promote muscle growth reducing body fat in food animals. CLB residues accumulate in meat and liver can produce adverse effects on humans, thus, the misuse of CLB must be controlled. An electrochemical immunosensor was described for the determination of CLB in pork meat using SPCEs modified with graphene sheets—Nafion films and core-shell Fe_3_O_4_@AuNPs. BSA-CLB biomagnetic conjugates were immobilized on the electrode and an indirect competitive immunoassay was performed with anti-CLB and free CLB/BSA-CLB. The electrochemical detection was carried out using Fe(CN)_6_^3−^ as the electroactive probe by measuring the decrease in the current signal as increases the steric hindrance produced by the formation of anti-CLB-CLB-BSA complex on the surface of SPCE. An increase in current proportional to the concentration of CLB (less CLA-BSA was immobilized) was observed over the range of 0.5 to 200 ng·mL^−1^ CLB, and a LOD value of 0.22 ng·mL^−1^ was obtained. Compared with other electrochemical immunosensors, this provided a better sensitivity but a narrower dynamic range. The immunosensor was employed to determine CLB in spiked pork meat [73].

A very similar strategy was also used by the same group for developing a voltammetric immunosensor for the determination of chloramphenicol (CAP). CAP is a broad-spectrum antibiotic used in veterinary medicine, which can cause serious toxic effects to humans. Although its application is banned in different countries, detection of CAP residues in food is still necessary. As in the previous protocol, SPCEs modified with graphene sheets-Nafion films and core-shell Fe_3_O_4_@AuNPs were employed, BSA-CAP biomagnetic conjugates were immobilized on the electrode, and an indirect competitive immunoassay was performed with anti-CAP and free CAP/BSA-CAP. The electrochemical detection by DPV using Fe(CN)_6_^3−^ provided a calibration plot over the range from 2.0 to 200 ng·mL^−1^ and a LOD of 0.82 ng·mL^−1^. The method was applied to spiked milk [74], and the results compared satisfactorily with those provided by liquid chromatography.

Fe_3_O_4_@AuNPs in combination with SPCEs were also used by Zhang et al. [75] to construct an immunosensor for detecting microcystin-(leucine-arginine) (MCLR), the most toxic species of the cyanotoxins. The capture antibody (anti-MCLR) was immobilized on the electrode surface by adsorption onto AuNPs, and the immunosensor utilized the direct competitive immunoassay format between the MCLR and HRP-MCLR. Electrochemical detection was made by DPV with H_2_O_2_ in the presence of HQ. Decrease in the peak current responses was proportionally related to MCLR level in the 0.79–12.9 ng·mL^−1^ concentration range. This result together with the LOD, 0.38 μg L^−1^, demonstrated the utility of the developed immunosensor for the analysis of water samples, as the WHO provisional guideline value is 1 μg·L^−1^. Noting that, by its characteristics, this immunosensor is competitive with chromatographic techniques recommended for MCLR determination. Furthermore, the reported method was applied to the analysis of tap and river spiked waters with good results.

Various natural and synthetic polymers have been used as modifiers of magnetic nanoparticles surface in order to take advantages of their attractive properties. Among them, electronic conducting polymers such as polyaniline (PANI) offer special interest due to the ease for preparing core-shell MNPs and their ability to immobilize biomolecules. PANI-coated γ-Fe_2_O_3_ were used to immobilize electrostatically a specific antibody for *Escherichia coli* O157:H7, and for the preparation of a sandwich-type electrochemical immunosensor using carbohydrate-capped AuNPs labeled with a polyclonal antibody. The electrochemical detection was performed by measuring the DPV oxidation signal from gold nanoparticles at a SPCE. A linear range of 10–10^6^ cfu·mL^−1^ and a LOD of 10 cfu·mL^−1^ were obtained [76].

PANI@γ-Fe_2_O_3_ MNPs were also used to fabricate an immunosensor for the determination of surface glycoprotein hemagglutinin (HA) from the Influenza A virus (FLUAV) H5N1(A/Vietnam/1203/04). Antibodies against target HA were immobilized onto MNPs and the resulting anti-HA–MNPs were shown to interact with glycans preincubated in mouse serum with HA. Glutaraldehyde, AuNPs and streptavidin-modified SPCEs were used to immobilize the biotinylated glycan/HA/anti-HA-MNPs complex. Cyclic volammetry was used to measure the charge transfer between PANI and the electrode surface, which significantly increased in the presence of target [77].

Another immunosensor was prepared by Pingarron´s group for the determination of *Legionella pneumophila* SG1 using SPCEs and MNPs prepared with dopamine (DA) electropolymerized on Fe_3_O_4_. After immobilization of a specific antibody (Ab) onto Fe_3_O_4_@pDA NPs, a sandwich immunoassay was carried out in the presence of bacteria using the HRP-labeled antibody (Ab-HRP). Once the resulting conjugates were captured on the electrode surface by applying a magnetic field, the amperometric response was measured after H_2_O_2_ addition in the presence of HQ. The LOD value achieved was 10^4^ cfu·mL^−1^ with no need for pre-concentration or pre-enrichment treatments. This method was applied to the analysis of water samples [78]. Relevant aspects of this work are the good stability during 30 days of the Ab-Fe_3_O_4_@pDA NPs and the possibility of detecting bacteria at 10 CFU·mL^−1^ level in less than three hours after a preconcentration step.

**Figure 5:**
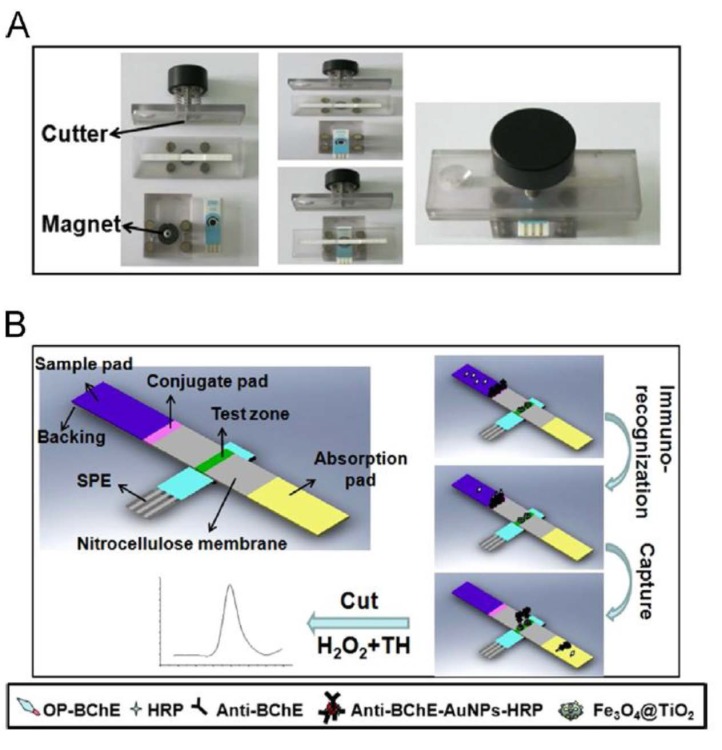
Schemes of (**A**) the system of portable phosphorylated butyrylcholinesterase (OP–BChE) test strip-based device; and (**B**) the detection principle. Reprinted from Ref. [80] with permission.

MNPs prepared by combination of iron oxides with other metal oxides possess improved properties for bioconjugation. Oxides that exhibit specific affinity to certain functional groups of interest are selected in this type of applications. For example, Fe_3_O_4_@TiO_2_ nanoparticles were used to develop two different configurations of electrochemical immunosensors for determining phosphorylated butyrylcholinesterase (OP-BChE), a biomarker of the exposure to organophosphorous pesticides (OP), in human plasma [79,80]. The magnetic nanomaterial was synthesized by a hydrothermal method and demonstrated a high selectivity for immobilizing the target OP-BChE by binding between the OP moiety and TiO_2_. Using this strategy, a first alternative consisted in the use of quantum dots (QDs)-tagged anti-BChE to prepare a sandwich-like complex, Fe_3_O_4_@TiO_2_/OP-BChE-anti-BChE-QDs, that can be magnetically isolated from the sample solutions and retained onto a SPCE. The cadmium from QDs can be detected by stripping SWV after platting the electrode surface with a film of bismuth. This immunosensor yielded a linear response for OP-BChE from 0.02 to 10 nM, with a LOD value of 0.01 nM [79]. More recently, the same group presented a disposable test-strip electrochemical immunosensor for determining OP–BChE where Fe_3_O_4_@TiO_2_ nanoparticles were adsorbed onto a test trip (Figure 5) and used to capture the target OP–BChE. Furthermore, recognition was made by using HRP and anti-BChE co-immobilized onto AuNPs. Once the sample solution containing OP-BChE migrates through the whole strip by capillary action, the test zone containing the immunocomplex is cutting to the SPCE cell, and detection by SWV is performed after addition of thionine and H_2_O_2_ [80].

Fe_3_O_4_@SiO_2_ nanoparticles constitute a variety of magnetic material with specific properties. Silica coating facilitates the dispersion of MNPs in aqueous solution by shielding the dipolar attraction between the nanoparticles and, moreover, it makes them more biocompatible and easier to bioconjugate. Ge et al. [81] used Fe_3_O_4_@SiO_2_ as signal labels in the preparation of an electrochemical immunosensor for microcystin-LR. A gold-nanoparticles-modified porous paper (Au-PW) was prepared resulting as interconnected AuNPs layers on the surface of cellulose fibers. Then, they were azide-functionalized using 1-azidoundecan-11-thiol and used as solid support to immobilize an alkyne-end-terminated antibody (Ab1) by a click reaction. Separately, azide-functionalized Fe_3_O_4_@SiO_2_NPs were prepared and used to immobilize both alkyne secondary antibody (Ab2) and alkyne-HRP. A sandwich-type immunoassay was employed and the electrochemical detection was carried out by placing the reaction zone of the Au-PW support on the active surface of a SPCE. The linear dependence ranged between 0.01 and 200 μg·mL^−1^, and the LOD value was 0.004 μg·mL^−1^.

**Figure 6:**
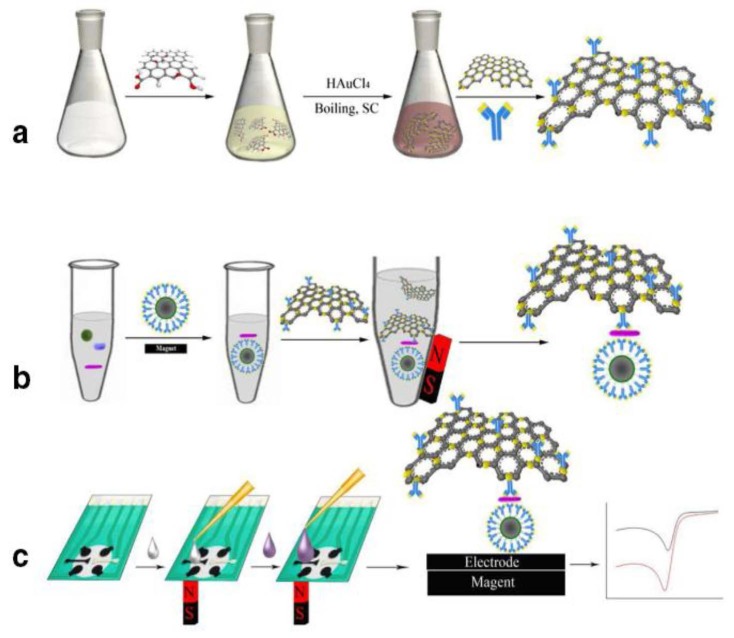
Fabrication of the electrochemical immunosensor for *Salmonella pullorum* and electrochemical detection: Synthesis process of rGO/Au/Ab2 (**a**); Capture of *Salmonella pullorum* from samples by MMPs (**b**); Put a MMPs-immunoconjugates drop on the SPCE and electrochemical detection (**c**). Reprinted from Ref. [82] with permission.

A sandwich electrochemical immunoassay for detecting *Salmonella pullorum* (Ag) was developed based on the use of Fe_3_O_4_/SiO_2_MNPs to immobilize the capture antibody (anti-*Salmonella pullorum*, Ab1), and reduced graphene oxide coated with gold nanoparticles (rGO/AuNPs) acting as the electrochemical label (Figure 6). Silica-coated MNPs were synthesized by reaction of previously obtained Fe_3_O_4_ with tetraethyl orthosilicate (TEOS) and 3-aminopropyl triethoxysilane (APTES) and, then, they were treated with glutaraldehyde for further Ab1 immobilization. A simple one-pot method was used to prepare rGO/AuNPs. Briefly, it consisted on mixing graphene oxide (GO) with HAuCl_4_, and boiled with sodium citrate. After capture the antigen, immobilized Fe_2_O_3_/SiO_2_Ab1/Ag onto a four-channels SPCE was sandwiched with rGO/AuNPs/Ab2. The electrochemical determination was performed by DPV after immersion of the immunosensor with the conjugate in HCl and preoxidation at +1.25 V vs. Ag/AgCl for 120 s. A linear relationship in the range of concentration from 10^2^ to 10^6^ cfu·mL^−1^ was found, with a LOD of 89 cfu·mL^−1^ [82].

The detection at trace level of HIV p24 antigen in serum could serve for promptly diagnosis of infection by human immunodeficiency virus (HIV). With this purpose, a disposable amperometric immunosensor was prepared using Fe_3_O_4_@AuNPs-coated MWCNTs to immobilize monoclonal anti-p24 antibody. SPCEs modified with *N*,*N*′-bis-(2-hydroxy-methylene)-o-phenylenediamine copper were used to retain the magnetic immunoconjugate, and the amount of p24 antigen was determined by measuring the decrease in the DPV cathodic peak current of the electroactive copper complex in the presence of H_2_O_2_ after the immunoreaction took place. A linear dependence with p24 concentration in the 0.6–160 ng·mL^−1^ range together with a LOD of 0.32 ng·L^−^^1^ were obtained. This approach was used to analyze serum samples of patients with AIDS, and the results obtained in the determination of p24 were in good agreement with those from ELISA method [83]. Some advantages of this configuration are the renewability of the electrode surface, and the use of the copper complex as the catalyst for H_2_O_2_ reduction instead HRP, with no need of redox mediator.

**Figure 7:**
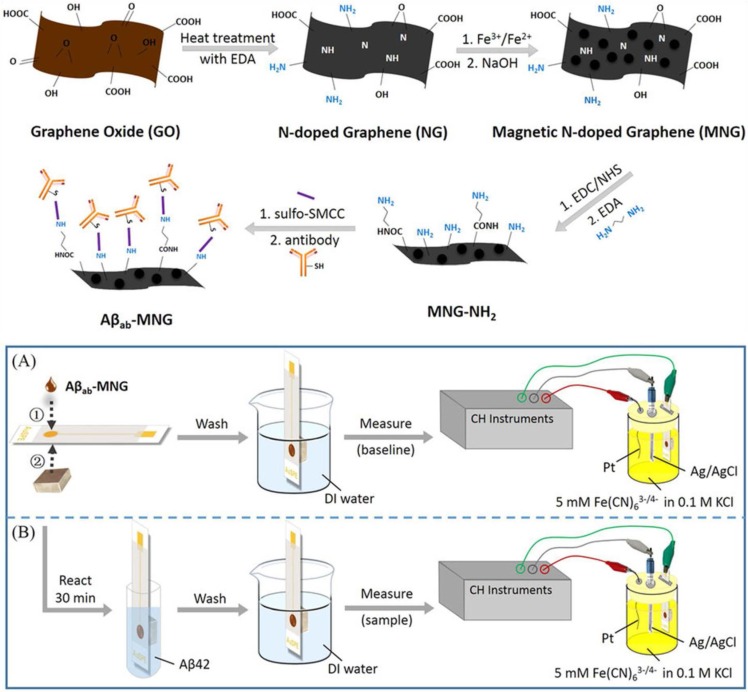
Graphical scheme representing preparation of magnetic immunoconjugates (Aβ_ab_-MNG), of the electrochemical detection by Aβ_ab_-MNG modified AuSPE (**A**) and the electrochemical detection of Aβ42 using Aβ_ab_-MNG modified AuSPE (**B**). Reprinted from Ref. [84] with permission.

A magnetic nitrogen-doped graphene-modified SPAuE with immobilized anti-Aβ 1–28 antibody was used to determine amyloid-beta peptide 1–42 (Aβ42), which is a reliable biomarker for Alzheimer disease´s early diagnosis. Chemical doping with nitrogen constitutes an effective approach to improve the electrical conductivity of graphene providing a better biocompatibility and sensitivity for biosensing applications. Fe_3_O_4_ NPs were deposited onto graphene doped with nitrogen to obtain a magnetic nanomaterial to be further labeled with anti-Aβ antibodies through a crosslinking method with sulfosuccinimidyl-4-(*N*-maleimidomethyl)cyclohexane-1-carboxylate (sulfo-SMCC). The magnetic immunosconjugates (Aβ_ab_-MNG) were deposited onto a screen-printed gold electrode Figure 7), and DPV in Fe(CN)_6_^2−/4−^ was used to register the calibration graphs. A linear plot within the range from 5 to 800 pg·mL^−1^ Aβ42 that covers the cut-off level of the antigen, with a LOD of 5 pg·mL^−1^, was obtained [84].

By analogy with MMPs, magnetic nanoparticles functionalized streptavidin in combination with SPEs have also been used. One example is the preparation of an immunosensor for detection of *Escherichia coli* O157:H7, one of the most dangerous foodborne pathogens. An impedimetric configuration was described involving immobilization of anti-*E. coli* onto Strept/Fe_3_O_4_. Bacterial cells from samples were isolated and concentrated onto MNBs and placed onto a screen-printed interdigitated electrode. Impedance measurements could detect *E. coli* O157:H7 within a linear range of 10^4^–10^7^ cfu·mL^−1^, with a LOD that corresponded to approximately 1400 cells in the used volume of 25 μL. This method demonstrated its utility by application to ground beef samples [85].

### 3.2. Magnetic Nanoparticles-Screen Printed Electrodes Electrochemical DNA Biosensors

MNPs also offer a potent tool for DNA-based electrochemical biosensing characterized by the low cost, high sensitivity and simple fabrication of the designed devices. One example is the disposable DNA biosensor using gold Fe_3_O_4_@AuNPs reported by Loaiza et al. [86] for detecting specific hybridization processes. Aβ_ab_-MNG/SPAuE was used to attach a thiolated 19-mer capture probe and, then, Strept-HRP was bound to the biotinylated target. The resulting magnetic complex was captured on the surface of a homemade SPCE, and the hybridization event was detected by SWV using hydroquinone as a mediator after the addition of H_2_O_2_. A low LOD (31 pM) was found for a 50-mer synthetic target without the need of PCR amplification.

**Figure 8:**
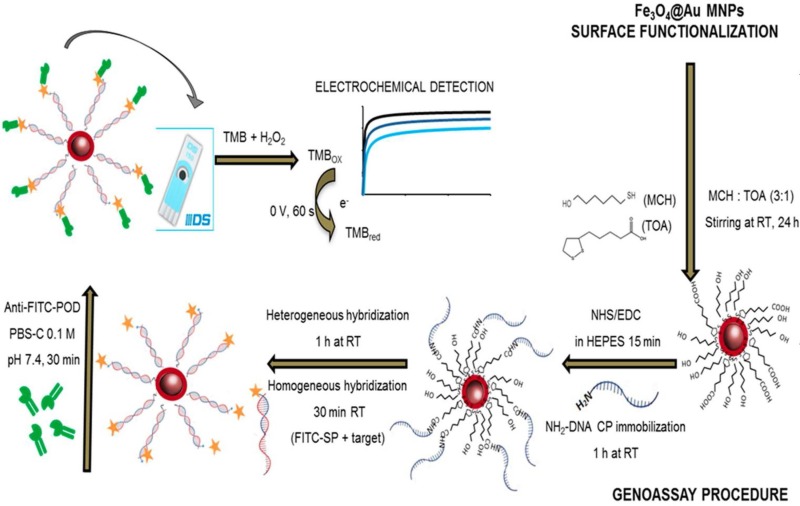
Scheme of the DNA electrochemical assay using Fe_3_O_4_@AuNPs for the detection of genetically modified organisms (GMOs). Adapted from Ref. [87] with permission.

Very recently, an electrochemical DNA assay using Fe_3_O_4_@AuNPs was developed for the detection of genetically modified organisms (GMOs) [87]. Core-shell MNPs were synthesized by coating iron oxide cores with a gold shell obtained through reduction of HAuCl_4_. The resulting particles were functionalized with a mixture of 6-mercapto-1-hexanol and thioctic acid in ethanol. A DNA probe was covalently linked to a carboxylated self-assembled monolayer, and a fluorescein isothiocyanate (FITC) detecting probe were also used in a sandwich assay format to recognize a specific fragment of the transgenic construct from MON810 maize. The use of anti-FITC-peroxidase Fab fragment conjugate as label, allowed chronoamperometric measurements (H_2_O_2_/TMB) of the enzyme activity captured on Fe_3_O_4_@AuNPs and placed on SPCEs, upon the hybridization event (Figure 8). This method provided a linear plot ranging between 0.25 and 2.5 nM, and a LOD of 0.15 nM, and demonstrated its analytical utility by application to certified transgenic samples measured after PCR amplification without further purification.

**Figure 9:**
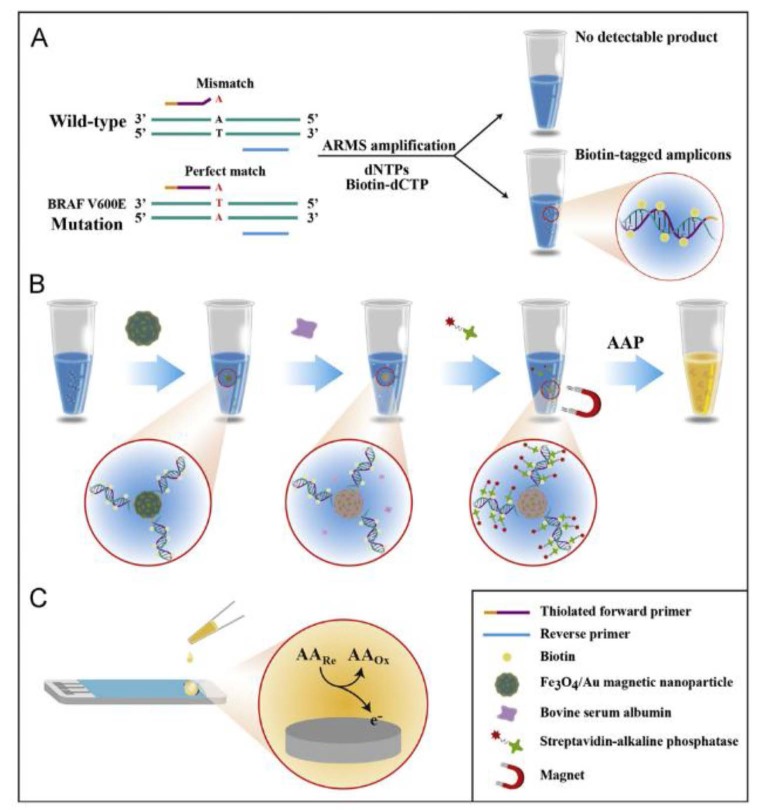
Scheme of the electrochemical assay of BRAF V600E mutation: (**A**) amplification of ARMS using thiol groups and biotinylated labels; (**B**) process for preparing multienzyme functionalized Fe_3_O_4_/AuNPs; (**C**) ascorbic acid oxidation at SPCE. Reprinted from Ref. [88] with permission.

The BRAF mutation is involved in causing cancers. An oncogenic (T→A) mutation at nucleotide 1799, which results in the substitution of valine with glutamate acid in codon 600 (V600E), is revealed as the most frequent of all BRAF genetic abnormalities [88]. A dual amplification strategy for detecting BRAFV600E mutation that combined the advantages of target enrichment by ARMS, that is amplification-refractory mutation system [89], and amplification of signals by enzymatic catalyzing reaction, has been reported. Once BRAF V600E alleles were selectively amplified, the products were tagged with biotinylated molecules by the incorporation of biotin-dCTP in the reaction. Fe_3_O_4_/AuNPs were used to capture the thiolated amplicons (Figure 9) and, after incubation with composite nanoparticles, Strept-AP were linked by biotin-streptavidin conjugation to DNA. Hence, the amount of AP loading on NPs was related to the amplicons quantity. The determination of mutant alleles was made by measuring the oxidation response of ascorbic acid on an SPCE using 2-phospho-L-ascorbic acid as the enzyme substrate. This method was applied to human colon adenocarcinoma cell lines where it shown to be capable of discriminating as few as 0.8% BRAF V600E DNA in the presence of an excess of wild-type background.

An electrochemical platform based on a chitosan/Fe_3_O_4_ biocomposite was also developed for electrochemical detection of HIV-1. A 25-mer gene expression of 91 modulator (GEM 91), which was capable of inhibit HIV-1 replication was covalently immobilized onto Chit/Fe_3_O_4_NPs by means of a phosphoramidate reaction between phosphate group at 5′ terminal of GEM sequence and amine group of CS. The hybridization process was detected by SWV with methylene blue (MB) as the redox indicator [90]. Pal et al. [91] used magnetite NPs coated with polyaniline for electrochemically detecting the anthrax Sterne strain (34F2) of *Bacillus anthracis*. An electrochemical sandwich assay was used consisting of a DNA detector probe labeled with PANI@γ-Fe_2_O_3_NPs and a biotinylated DNA capture probe. Once hybridization with DNA targets took place, the Fe_3_O_4_@PANI/detector probe–DNA/capture probe–biotin hybrid was magnetically immobilized onto a Strept-modified SPCE. The electrochemical detection was made by measuring the characteristic voltammetric peaks of polyaniline from PANI@γ-Fe_2_O_3_NPs. The LOD was 0.01 ng μL^−1^ DNA.

**Figure 10:**
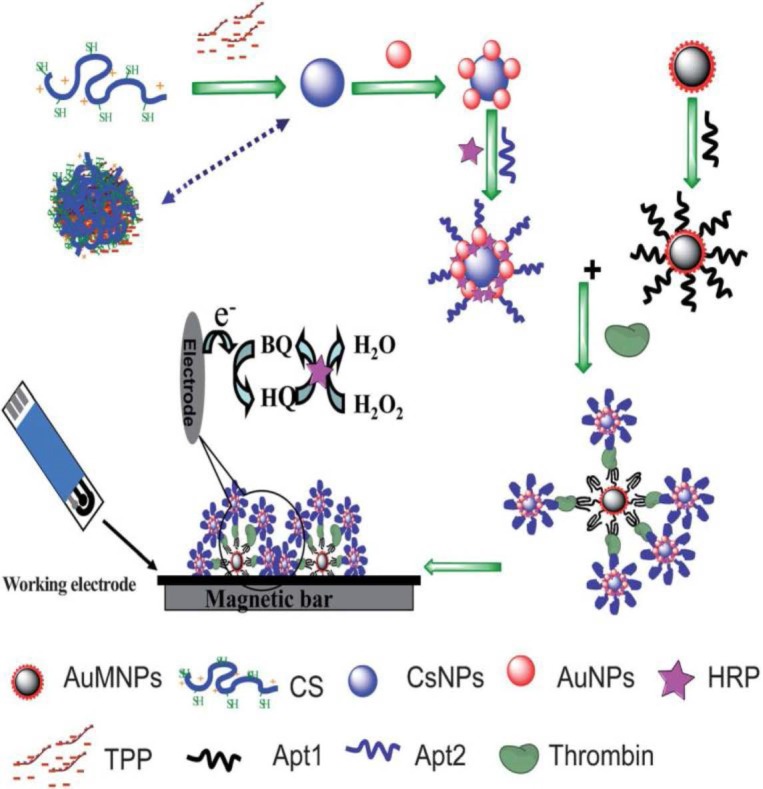
Scheme of the preparation of aptasensor for thrombin detection. Reprinted from Ref. [92] with permission.

Zhao et al. [92] developed a highly selective and sensitive voltammetric aptasensor for the determination of thrombin by using core–shell Fe_3_O_4_–AuNPs to immobilize an anti-thrombin aptamer (Apt1). Separately, chitosan–AuNPs and HRP conjugates (CS–AuNPs–HRP) were used to immobilize another anti-thrombin aptamer (Apt2) for signal amplification. A sandwich MNPs–Apt1/thrombin/Apt2–CS–AuNPs–HRP structure was formed in the presence of thrombin that was further captured onto a SPCE (Figure 10). The abundant HRP present on the electrode surface dramatically accelerated the oxidation of HQ with H_2_O_2_, this providing DPV currents which could be linearly correlated to the concentration of thrombin in the 0.01–10 pM range with a LOD of 5.5 fM.

### 3.3. Magnetic Nanoparticles as Labels for Signal Amplification

In addition to their utility for preparing bioconjugates easily captured on the electrode surfaces, other interesting applications of MNPs rely as signal amplification labels. Ahmadi et al. [93] used Fe_3_O_4_@AuNPs as electrochemical labels in the construction of an electrochemical immunosensor for digoxin. Digoxin is a drug that helps the heart pumps better which a high concentration can be toxic. A competitive configuration was developed using a secondary antibody immobilized onto a polyvinylalcohol (PVA)-modified SPCE surface, as well as BSA-digoxin conjugates immobilized onto MNPs, and anti-digoxin antibody. DPV was employed for quantitative detection of digoxin in serum after immersing the electrode in HCl for the electrochemical oxidation of gold, and further voltammetric reduction of the generated AuCl_4_^−^. This strategy allowed determining digoxin within the range from 0.5 to 5 ng·mL^−1^ with a LOD of 0.05 ng·mL^−1^.

**Figure 11:**
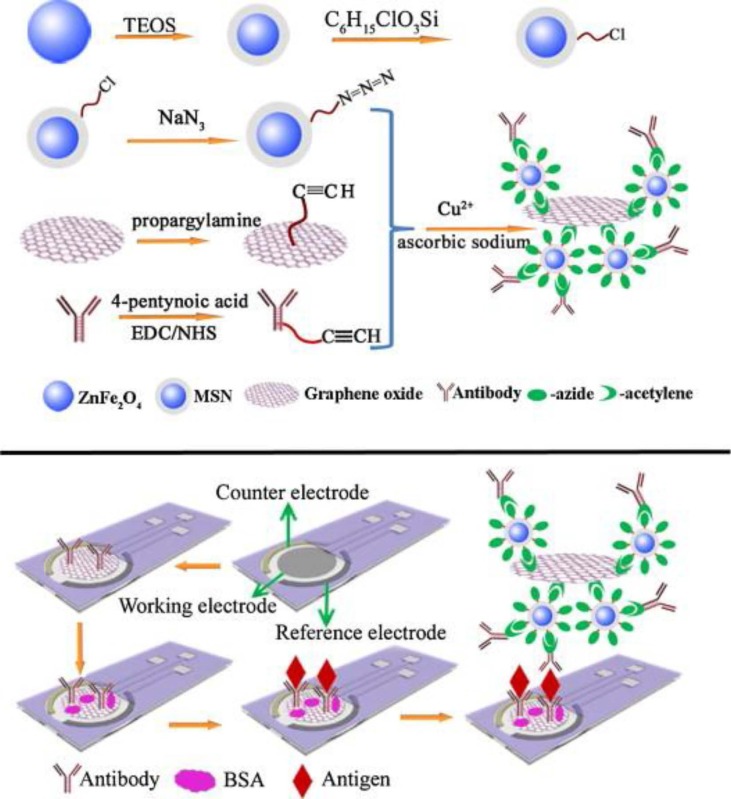
Disposable immunosensor for detection of cancer antigen 153 using MSNs/GO-Ab2 conjugates as advanced labels. Reprinted from Ref. [94] with permission.

ZnFe_2_O_4_ magnetic nanoparticles possess intrinsic peroxidase-like activity, also exhibiting good catalytic properties, stability, and dispersibility, among other advantages [94]. Silica coated-ZnFe_2_O_4_ (MSNs) were used to prepare a disposable immunosensor for detection of cancer antigen 153. “Click” chemistry was employed to conjugate the azide-functionalized MSNs to alkynylated-GO, and the resulting enzyme-like MSNs/GO was used as label for signal amplification after immobilizing a detector anti-CA153 antibody (Ab2) (Figure 11). A sandwich assay was used by immobilization of a monoclonal anti-CA153 capture antibody (Ab1) to a GO-modified SPCE and conjugation of CA153-Ab1-GO/SPCE with MSNs/GO-Ab2 labels. The electrochemical measurements were made by DPV upon H_2_O_2_ addition in the presence of thionine as the redox mediator. Peak currents were proportionally related to the logarithm value of CA 153 concentration between 10^−3^ to 200 U·mL^−1^, and the LOD achieved was 2.8 × 10^−4^ U·mL^−1^. Validation of this immunosensor was made by application to the analysis of spiked serum samples.

### 3.4. Multiplexing Using Magnetic Nanoparticles-Screen Printed Electrodes

**Figure 12:**
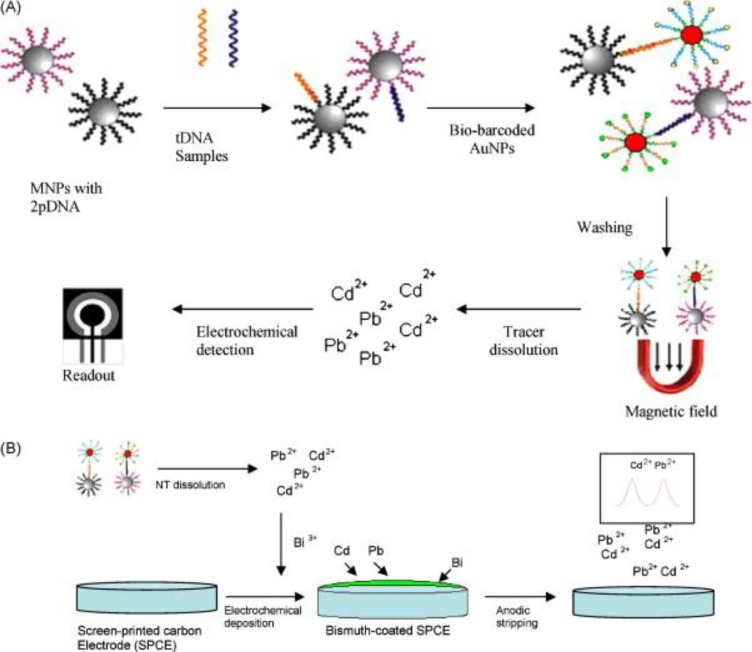
Schematic display of: (**A**) bio-barcode assay for the detection of protective antigen A (*pagA*) gene of *B. anthracis* and insertion element (*Iel*) gene of *S. enteritidis*; and (**B**) square-wave anodic stripping voltammetry. Reprinted from Ref. [95] with permission.

The simultaneous multiple detection of the protective antigen A (*pagA*) gene of *Bacillus anthracis* and the insertion element (*Iel*) gene of *Salmonella enteritidis* was performed by using a bio-barcoded electrochemical biosensor. AuNPs and MNPs were coated, respectively, with two target-specific DNA probes: 1pDNA, which can recognize one end of the target DNA sequence (tDNA), and 2pDNA, that can recognize the other end of the target gene. PbS and CdS were also utilized as nanoparticle tracers (NTs) to prepare NTs-terminated bio-barcode ssDNA (bDNA-NTs) which acted as signal reporters and amplifiers. After binding the nanoparticles with the target DNA, a sandwich MNP-2pDNA/tDNA/1pDNA-AuNP-bDNA-NTs was prepared. Once the nanoparticle tracer was dissolved in nitric acid, the NT^2+^ ions were detected by SWASV onto SPCE (Figure 12). Due to the large amount of nanoparticle tracers per AuNPs-DNA probe, a substantial amplification could be attained, the detection limits being of 0.5 ng·mL^−1^ for *Iel* gene of *S. enteritidis,*(using CdS), and 50 pg·mL^−1^ for *pagA* gene of *B. anthracis* (using PbS) [95].

## 4. General Conclusions and Future Prospects

This review article sheds useful insights into the unique opportunities offered by magnetic particles coupled to disposable electrochemical platforms in the design of very sensitive electroanalytical biosensors and bioassays. Approaches highlighted and discussed above demonstrate the high utility of magnetic particles to get an amplified electrochemical transduction of biomolecular recognition events using as transducer modifiers, carriers or advanced labels.

The highly-sensitive biodetection schemes highlighted open up the possibility of detecting a wide spectrum of relevant analytes (clinical biomarkers, bacterial pathogens, antimicriobials, drugs, infectious agents, food allergens) and in different fields. Approaches selected demonstrated also the compatibility of magnetic materials funtionalization with a variety of recognition probes (e.g., antibodies, aptamers, DNA probes, viral proteins, etc.), the extensively use of SPCEs as disposable trasnsducers and the wide number of assay formats (sandwich, direct and competitive) and electrochemical techniques ((chrono)amperometry, DPV, SWV, SWASV and LSV) employed.

All these protocols have demonstrated to be advantageous in terms of sensitivity, simplicity, and assay time compared to the state of the art ELISA and PCR-based methodologies. Although preparation of the magnetic bioconjugates usually requires long protocols, they can be prepared in advance and stored until the determinations should be performed, therefore allowing shorter assay times (usually 1–2 h). Other advantages related to the use of electrochemical transduction included the use of portable and cost-effective instrumentation, able to perform multiplexed detection which can be claimed as important practical advantages in the implementation of user-friendly devices for decentralized and routine analysis.

Although MMPs and MNPs have demonstrated to improve significantly the analytical performance of electrochemical affinity biosensors, comparative studies demonstrated that. MNPs have larger surface-to-volume ratio providing more chemical reactive sites for the attachment of higher amounts of smaller size (<1 μm) biomolecules at their surface. Despite this improved reactivity, similar or slightly better analytical performance with MNPs compared to MMPs has been reported in standard solutions [96]. However lower efficiency for immunomagnetic separation was achieved in complex samples (such as whole milk) using MNPs attributable to these nanoparticles might be more affected by matrix effect [5]. Moreover, MNPs are easily aggregated and required longer times for magnetic actuation [96]. The wide commercial availability of MMPs modified with various functional groups greatly facilitates the design of many different approaches test depending on the particular application. Moreover, although the bigger size of the MMPs can pose some limitations in the final sensitivity of the approach because the labeling reagent was not in direct contact with the electrode surface [1], currently the use of small magnets below the surface of the working electrode of the SPEs allows to concentrate the MBs on the electrode surface and minimize this effect. An additional advantage of MNPs is the easy modification by coating with different materials to give hybrid nanoparticles that can be used for further functionalization with specific components such as catalytically active species [97]. Furthermore, MNPs can also be utilized as signal reporters; Fe_3_O_4_ nanoparticles have shown peroxidase-like activity [98] and can be used as artificial enzymes for signal amplification. In this sense, although the use of magnetic materials-bioconjugates as label for biosensing has been much less explored that their use to modify transducers, lessons learned in the few reported approaches using MNPs (no examples found using MMPs) will provide useful starting points in this direction. It is worth mentioning also that the selected examples demonstrated that while commercial MMPs are used generally without further modification apart from the bioreceptor immobilization, the MNPs offer a greater versatility for modification. Apart from the common Au core/shell modification, MNPs have been modified with natural and synthetic polymers (PANI, DA), other metal oxides (TiO_2_, SiO_2_) and nanomaterials (MWCNTs).

It is also worth mentioning that in order to increase even more the sensitivity of these magnetic particles-based disposable electrochemical bioplatforms some approaches based on their coupling with nanomaterial-based signal or target amplification strategies have been described also.

Although electrochemical disposable electrochemical biosensors using magnetic particles have demonstrated very attractive advantages for enhancing and superseding the capabilities of current analytical methodologies mainly in terms of simplicity, assay time and analysis of complex samples, this field, especially with MNPs, is still new and there are many points to be addressed.

Further research is required for the development of protocols for synthesis, functionalization and bioconjugation of MNPs with high level of uniformity and reproducibility to avoid significant variation in measurements. Moreover, to broaden the scope of bioapplications and open new alternatives, the construction of new hybrid magnetic materials with ideal building blocks such as functional nanoparticles or biomolecules is also highly desirable.

Other important challenge is testing with real applications. Although in most of cases very low LODs are achieved improving those obtained with other conventional approaches, a big portion of the reports deal with pure analytes in buffer solutions. Therefore, researchers should also make more effort on applying these attractive designs to the analysis of complex biological matrices and solve the problem of MNPs aggregation in these media. Validation tests to demonstrate the applicability, trueness and selectivity in real samples are also required. In this context, simplified protocols able to decrease the time required for the assay would help an easier and wider applicability.

Moreover, most of these biosensor devices still remain in the proof of concept or prototype stages of development and their transfer to practical applications is a challenging process which so far has rarely resulted in market implementation. Taking into account the disposable nature of the electrochemical transducers used, it will be very helpful also the implementation of these magnetic bioassays in microfluidic systems allowing the determination to be performed with minimal manipulation. Solving drawbacks associated with non-specific binding, the reproducibility of the measurements, and the long-term stability of the developed devices are also needed.

Although some approaches for dual detection have been reported, the simultaneous determination of multiple targets in complex samples still remains unsolved and the design of novel approaches for multiplexed analysis of a large number of analytes needs yet to be explored.

Despite these challenging issues which remain to be addressed, taking into account the fast advancement and great advantages offered by electrochemical biosensors using disposable electrochemical platforms and MMPs or MNPs-bioconjugates to develop sensitive, simple and fast detection systems that may provide robust tools for biosensor research, it is expected that these approaches will reach an increasing importance within the electroanalytical research area in the near future in diverse scientific fields such as in quantification of biotargets, and early detection of genetic mutations or diseases.

## Supplementary Materials

The following are available online at http://www.mdpi.com/1424-8220/16/10/1585/s1, Table S1, Table S2 and Table S3.
