# Supplementary material for: Magnetic Particles Coupled to Disposable Screen Printed Transducers for Electrochemical Biosensing
Source: Sensors (Basel). 2016 Sep 25;16(10):1585. doi: 10.3390/s16101585 (PMC5087374; doi:10.3390/s16101585)
Supplement: Supplementary file 1 [file sensors-16-01585-s001.docx]

Supplementary Materials: Magnetic Particles Coupled to Disposable Screen Printed Transducers for Electrochemical Biosensing

Paloma Yáñez-Sedeño, Susana Campuzano and José M. Pingarrón

**SUPPORTING INFORMATION**

| CONTENTS | PAGE |
| --- | --- |
| Table S1 | S2–S4 |
| Table S1 | S5 |
| Table S1 | S6 |

**Table S1.** MMPs-based electrochemical immunosensing approaches.

| **Electrodes** | **Type of MMPs** | **Biomarker** | **Format** | **Electrochemical Technique** | **L.R.** | **LOD** | **Immunosensor Fabrication/Assay Time** | **Reference** |
| --- | --- | --- | --- | --- | --- | --- | --- | --- |
| SPCEs | ProtA-MMPs | Cortisol | Direct competitve | DPV | 5.0 × 10^−3^–150 ng·mL^−1^ | 3.5 pg·mL^−1^ | 30 min/53 min | [11] |
| SPCEs | ProtA-MMPs | Testosterone | Direct competitive | Amperometry | 5.0 × 10^−3^–50 ng·mL^−1^ | 1.7 pg·mL^−1^ | 10 min/45 min | [12] |
| Au-SPEs | ProtA-MMPs | *Streptococcus pneumoniae* | Sandwich | Amperometry | 4.3 × 10^4^–1.0 × 10^7^ (capsulated strain) | 1.5 × 10^4^(capsulated strain) | 1 h/2 h | [13] |
|  |  |  |  |  | 7.5 × 10^5^ to | 6.3 × 10^5^ |  |  |
|  |  |  |  |  | 1.0 × 10^8^ (non-encapsulated strain) cfu·mL^−1^ | (non-encapsulated strain) cfu·mL^−1^ |  |  |
| SPCEs | Tosyl activated-MMPs | Myoglobin | Sandwich | Steps and sweep voltammetry | 0.2–20 ng·mL^−1^ (10 pM–1 nM) | — | 2 h/40 min | [14] |
| SPCEs | Strep-MMPs | PRL | Sandwich | DPV | 10 and 2000 ng·mL^−1^ | 3.74 ng·mL^−1^ | 45 min/1 h 8 min | [15] |
| Au-SPEs | Tosyl activated-MMPs | hGH | Sandwich | SWV | 0.01 and 100 ng·mL^−1^ | 0.005 ng·mL^−1^ | 19 h/2 h 15 min | [16] |
| SPCEs | ProtG-MMPs | *Phakopsora pachyrhizi* | Sandwich | DPV | 5.0 to 45.0 μg·mL^−1^ | 18 ng·mL^−1^ | 30 min/1 h 30 min | [17] |
| SPCEs | HOOC-MMPs | D-dimer | Indirect competitive and sandwich | Amperometry | 0.084–1.9 (indirect competitive) 0.06–1.0 μg·mL^−1^ (sandwich) | 0.028 (indirect competitive) and 0.020 μg·mL^−1^ (sandwich) | 2 h 55 min/30 min (indirect competitive) | [18] |
|  |  |  |  |  |  |  | 2 h 25 min/1 h (sandwich assay) |  |
| Au-SPEs | ProtA-MMPs | Staphylococcal protein A (ProtA) and *Staphylococcus aureus* | Direct competitive | Amperometry | 1.7 × 10^−8^–2.6 × 10^−6^μg·mL^−1^ (ProtA) | 3.9 × 10^−9^μg·mL^−1^ (ProtA), 1 cfu *S. aureus* mL^−1^ (in raw milk samples) | 30 min/1 h | [19] |
|  |  |  |  |  | 1.0–1.0 × 10^7^ (lysed *S. aureus* cells) |  |  |  |
| SPCEs | ProtG-MMPs | TCs | Direct competitive | Amperometry | 12.5–676.2 ng·mL^−1^ (TC) | 3.9 ng·mL^−1^ (TC) | 30 min/30 min | [20] |
| SPCEs | Strep-MMPs | LPO | Direct | Amperometry | 0.42–12.5 mg·mL^−1^ | 0.12 mg·mL^−1^ | 15 min/30 min | [21] |
| Strip of 8 graphite SPEs | Tosyl activated-MMPs | anti-IgG-HRP | Direct | Amperometry | — | — | 20 h 30 min/30 min | [22] |
| Graphite SPEs | ProtG-MMPs | MUC1 | Sandwich | DPV | 0–25 ppb | 1.34 ppb | 2 h/3 h 30 min | [23] |
| Electrodes | Type of MMPs | Biomarker | Format | Electrochemical technique | L.R. | LOD | Immunosensor fabrication/assay time | Reference |
| SPCEs | Tosyl activated-MMPs | ATG2 | Indirect | SWV | 1:10,300–1:3000 | 1:14,200 | 30 h/1 h | [24] |
| SPCEs | ProtA-MMPs, Strep-MMPs | Cp | Direct competitive | DPV | 0.1–1000 mg·mL^−1^ (Prot A-MMPs) | 0.040 mg·mL^−1^ (Prot A-MMPs) | 15 min/35 min (both types of MMPs) | [25] |
|  |  |  |  |  | 0.025–20 mg·mL^−1^ (Strept-MMPs) | 0.018 mg·mL^−1^ (Strept-MMPs) |  |  |
| Au-SPEs | HOOC-MMPs | CRP | Sandwich | Amperometry | 0.07–1000 ng·mL^−1^ | 0.021 ng·mL^−1^ | 2 h 55 min/1 h 30 min | [26] |
| Au-SPEs | Strep-MMPs | cTnT | Sandwich | Amperometry | 0.05–1.0 ng·mL^−1^ | 0.017 ng·mL^−1^ | 45 min/1 h 15 min | [27] |
| Au-SPEs | HOOC-MMPs | NT-proBNP | Indirect competitive | Amperometry | 0.12–42.9 ng·mL^−1^ (10-times diluted serum) | 0.02 ng·mL^−1^ (10-times diluted serum) | 2 h 55 min/1 h 15 min | [28] |
| SPCEs | HOOC-MMPs | IL-6 | Sandwich | Amperometry | 1.75–500 pg·mL^−1^ | 0.39 pg·mL^−1^ | 2 h 25 min/1 h 30 min | [29] |
| SPCEs | His-Tag-Isolation-MMPs (Direct),  HOOC-MMPs (Indirect) | Fibrinogen | Direct and indirect competitive | Amperometry | 0.5–250.0 μg·mL^−1^ (Direct) | 0.49μg·mL^−1^ (Direct) | 10 min/1 h 30 min (Direct) | [30] |
|  |  |  |  |  | 0.15–6.18 μg·mL^−1^ (Indirect) | 0.044 μg·mL^−1^ (Indirect) | 2 h 55 min/1 h 30 min (Indirect) |  |
| SPCEs | Strep-MMPs | Fibrinogen | Indirect competitive | Amperometry | 0.004–0.8 µg·mL^−1^ | 0.8 ng·mL^−1^ | 30 min/30 min | [31] |
| SPCEs | HOOC-MMPs | TNFα | Sandwich | Amperometry | — | 2.0 pg·mL^−1^ (standard solutions), 5.8 pg·mL^−1^ (human serum) | 2 h 40 min/2 h 10 min | [32] |
| SPCEs | HOOC-MMPs | ErbB2 | Sandwich | Amperometry | 0.1–32.0 ng·mL^−1^ | 26 pg·mL^−1^ | 2 h 40 min/1 h | [33] |
| SPCEs | ProtG-MMPs | GHRL | Direct competitive | DPV | 10^−3^–10^3^ ng·mL^−1^ | 7 pg·mL^−1^ | 1 h/1 h 20 min | [34] |
| SPCEs | ProtG-MMPs | OTA | Direct competitive | Amperometry | 1.3–153.8 μg L^−1^ | 0.32 μg L^−1^ | 2 h/45min | [35] |
| SPCEs | ProtG-MMPs | FB1, FB2, FB3 | Direct competitive | Amperometry | 0.73–11.2 μg L^−1^ (FB1) | 0.33 μg L^−1^ (FB1) | 2 h/1 h | [36] |
| SPCEs | HOOC-MMPs | Lp(a) | Sandwich | Amperometry | 0.01–0.5 μg·mL^−1^ | 4 ng·mL^−1^ | 2 h 10 min/15 min | [37] |
| SPCEs | ProtG-MMPs | APAP | Direct | DPV | 5.28 μM–0.75 mM | 1.76 μM | 45 min/10 min | [38] |
| Pt-SPEs | HOOC-MMPs | OVA | Sandwich | LSV | 11–222 nM | 5 nM | 12 h 30 min/2 h | [39] |
| SPCEs | HOOC-MMPs | Ara h 1 | Sandwich | Amperometry | 20.8–1000.0 ng·mL^−1^ | 6.3 ng·mL^−1^ | 2 h 25 min/2 h | [40] |
| SPCEs | HOOC-MMPs | Ara h 2 | Sandwich | Amperometry | 87–10,000 pg·mL^−1^ | 26 pg·mL^−1^ | 2 h 10 min/1 h 15 min | [41] |
| SPCEs | HOOC-MMPs | β-LG | Sandwich | Amperometry | 2.8–100 ng·mL^−1^ | 0.8 ng·mL^−1^ | 2 h 25 min/1 h | [42] |
| Electrodes | Type of MMPs | Biomarker | Format | Electrochemical technique | L.R. | LOD | Immunosensor fabrication/assay time | Reference |
| SPCEs | HOOC-MMPs | α-LA | Sandwich | Amperometry | 37.0–5000 pg·mL^−1^ | 11.0 pg·mL^−1^ | 2 h 10 min/30 min | [43] |
| SPCEs | HOOC-MMPs | ERα | Sandwich | Amperometry | 63–2000 pg·mL^−1^ | 19 pg·mL^−1^ | 2 h 45 min/2 h 10 min | [44] |
| Array of 8 SPCEs | ProtG-MMPs | COC | Direct competitive | Amperometry | — | 0.09 (PBS), 0.36 (urine), 0.09 (saliva), and 0.63 (human serum) ng·mL^−1^ | 10 min/90 min | [9] |
| SPCEs | ProtG-MMPs | miRNA-205 | Direct | Amperometry | 8.2–250 pM | 2.4 pM | 45 min/2 h | [45] |
| SPCEs | HOOC-MMPs | Endoglin | Sandwich | Amperometry | 0.8–10.0 ng·mL^−1^ | 0.2 ng·mL^−1^ | 2 h 10 min/30 min | [46] |

APAP: acetaminophen; ATG2: anti-transglutaminase antibodies; β-LG: β-lactoglobulin; cfu: colony forming unit; COC: cocaine; Cp: ceruloplasmin; CRP: human C-reactive protein; cTnT: human cardiac troponin T; DPV: differential pulse voltammetry; ERα: estrogen receptor α; FB1: fumonisin B1; FB2: fumonisin B2; FB3: fumonisin B3; GHRL: ghrelin; hGH: human growth hormone; IL-6: interleukine-6; α-LA: α-lactalbumin; OTA: ochratoxin A; LPO: lactoperoxidase; OVA: ovalbumin; Lp(a): lipoprotein(a); LSV: linear sweep voltammetry; NT-proBNP: amino-terminal pro-B-type natriuretic peptide; PRL: prolactin; SPCE: screen-printed carbon electrodes; SWV: square-wave voltammetry; TCs: tetracyclines; TNFα: tumor necrosis factor alpha.

**Table S2.** MMPs-based electrochemical genosensing approaches.

| **Electrodes** | **Type of MMPs** | **Biomarker** | **Format** | **Electrochemical Technique** | **L.R.** | **LOD** | **Genosensor Fabrication/Assay Time** | **Reference** |
| --- | --- | --- | --- | --- | --- | --- | --- | --- |
| Au-SPEs | Strep-MMPs | *S. pneumoniae* | Direct (+daPCR) | Amperometry | 5.0–36.0 nM (synthetic target)  1.0–6.0-nM (aPCR amplicon) | 5.1 nM (synthetic target)  1.1 nM (aPCR amplicon)  2 cfus (daPCR) | 1 h/2 h 30 min (without aPCR)  1 h/4 h 22 min (with DaPCR) | [47] |
| MUX-SPCE16s | Strep-MMPs | miRNA-15a | Direct | LSV | 2.5–10.0 μg·mL^−1^ | 0.114 μg·mL^−1^ | 15 min/40 min | [49] |
| SPCEs | Chitin-MMPs | miRNA-21 | Direct | Amperometry | 0.14–10.0 nM | 0.04 nM | 20 min/1 h 45 min | [50] |
| SPCEs | Strep-MMPs | miRNA-21 | Direct | Amperometry | 1.4–10.0 nM | 0.42 nM | —/1 h | [51] |
| SPCEs | HOOC-MMPs | miRNA-21 | Direct+ISDPR | DPV | 10 fM–10 nM | 9 fM | 12 h 30 min/2 h 30 min | [4] |
| SPCEs | Strep-MMPs | miRNA-21 | Sandwich+HCR | Amperometry | 0.2–5.0 nM | 60 pM | 1 h/45 min | [52] |

aPCR: asymmetric PCR; daPCR: direct aPCR;MUX-SPCE16s: multi-channel screen-printed array of 16 carbon electrodes; HCR: hybridization chain reaction; ISDPR: isothermal strand-displacement polymerase reaction.

**Table S3.** MMPs-based electrochemical multiplexed approaches.

| **Electrodes** | **Type of MMPs** | **Biomarker** | **Format** | **Electrochemical Technique** | **L.R.** | **LOD** | **Genosensor Fabrication/Assay Time** | **Reference** |
| --- | --- | --- | --- | --- | --- | --- | --- | --- |
| MUX-SPCE16s | Strep-MMPs | miRNA-16, miRNA-15a and miRNA-660 | Direct | DPV | 5–80 μg·mL^−1^ | 4.3 pmole in 3 μL sample | 15 min/15 min | [55] |
| SPdCEs | HOOC-MMPs | CRP and NT-proBNP | Sandwich (CRP) and indirect competitive (NT-proBNP) | Amperometry | 2.0–100 ng·mL^−1^ (CRP)  2.5 to 504 ng·mL^−1^ (NT-proBNP) | 0.47 ng·mL^−1^ (both biomarkers) | 2 h 55 min/1 h 15 min | [56] |
| SPCEs | ProtG-MMPs and His-tag isolation-MMPs | CPHs, SAs and TCs | Direct competitive | Amperometry | — | — | 30 min/5 min | [57] |
| SPCEs | Strep-MMPs | Fragments of Lec and RR genes | Direct | Chronoamperometry (lectin) and DPV (RRS) | 2–250 pM (both targets) | 190 (lectin) and 650 fM (RRS) | 30 min/2 h 15 min | [58] |
| SPdCEs | Chitin-MMPs | miRNA-21 and miRNA-205 | Direct | Amperometry | 2.0–10.0 nM | 0.6 nM | 20 min/2 h | [59] |
| SPdCEs | HOOC-MMPs | ERα and PR | Sandwich | Amperometry | 73–1,500 (PR) and 63–2000 pg·mL^−1^ (ERα) at SPCEs | 22 (PR) and 19 (ERα) pg·mL^−1^ at SPCEs | 2 h 45 min/2 h 10 min | [60] |
| SPdCEs | HOOC-MMPs (protein) and Strep-MMPs (mRNA) | IL-8 protein and IL-8 mRNA | Sandwich (protein) and direct (mRNA) | Amperometry | 241.3–5000 pg·mL^−1^ (protein)  0.69–7.5 nM (mRNA) | 72.4 pg·mL^−1^ (protein),  0.69 nM (mRNA) | 3 h/1 h 45 min (protein)  1 h/45 min (mRNA) | [61] |
| SPdCEs | HOOC-MMPs | Ara h 1 and Ara h 2 | Sandwich | Amperometry | 60–1000 (Ara h 1) and 0.25–5 (Ara h 2) ng·mL^−1^ | 18.0 (Ara h 1) and 0.07 (Ara h 2) ng·mL^−1^ | 2 h 25 min/2 h | [62] |

CPHs: cephalosporins; CRP: C-reactive protein; ERα: estrogen receptor α; MUX-SPCE16s: multi-channel screen-printed array of 16 carbon electrodes; NT-proBNP: amino-terminal pro-B-type natriuretic peptide; PR: progesterone receptor; SAs: sulfonamides; SPdCEs: screen-printed dual carbon electrodes; TCs: tetracyclines.
